# 10 Years of SYNTAX: Closing an Era of Clinical Research After Identifying New Outcome Determinants

**DOI:** 10.1016/j.jacasi.2023.03.014

**Published:** 2023-05-30

**Authors:** Patrick W. Serruys, Pruthvi C. Revaiah, Kai Ninomiya, Shinichiro Masuda, Nozomi Kotoku, Shigetaka Kageyama, Yoshinobu Onuma, Marie Angele Morel, Scot Garg, Ted Feldman, Arie Pieter Kappetein, David R. Holmes, Michael J. Mack, Friedrich-Wilhelm Mohr

**Affiliations:** a Department of Cardiology, National University of Ireland, Galway (NUIG), and CORRIB Research Centre for Advanced Imaging and Core Laboratory, Galway, Ireland; b Department of Cardiology, Royal Blackburn Hospital, Blackburn, United Kingdom; c Edwards Lifesciences, Irvine, California, USA; d Department of Cardiothoracic Surgery, Erasmus University Medical Centre, Rotterdam, the Netherlands; e Department of Cardiovascular Diseases and Internal Medicine, Mayo Clinic, Rochester, Minnesota, USA; f Department of Cardiothoracic Surgery, Baylor Scott & White Health, Dallas, Texas, USA; g University Department of Cardiac Surgery, Heart Centre Leipzig, Leipzig, Germany

**Keywords:** CABG, coronary artery bypass grafting, machine learning, PCI, percutaneous coronary intervention, SYNTAX

## Abstract

The SYNTAX trial randomized patients equally eligible for coronary artery bypass grafting or percutaneous coronary intervention using the Heart Team approach. The SYNTAXES study achieved a follow-up rate of 93.8% and reported the 10-year vital status. Factors associated with increased mortality at 10 years were pharmacologically treated diabetes mellitus, increased waist circumference, reduced left ventricular function, prior cerebrovascular and peripheral vascular disease, western Europe and North American descent, current smoking, chronic obstructive pulmonary disease, elevated C-reactive protein, anemia, and an increase in HbA1c. Procedural factors associated with higher 10 years mortality include periprocedural myocardial infarction, extensive stenting, small stents, ≥1 heavily calcified lesion, ≥1 bifurcation lesion, residual SYNTAX score >8, and staged percutaneous coronary intervention. Optimal medical therapy at 5 years, use of statins, on-pump coronary artery bypass grafting, multiple arterial grafts, and higher physical component score and mental component score were associated with lower mortality at 10 years. Numerous scores and prediction models were developed to help individualize risk assessment. Machine learning has emerged as a novel approach for developing risk models.

## Highlights

• Risk stratification among patients with complex CAD is central to decision making between CABG and PCI.
• Revascularization strategy in complex CAD should consider multiple factors (clinical, procedural, postprocedural) and analysis includes interaction between these multiple factors.
• We have seen evolution of risk factor assessment from conventional multivariable models to models based on the interaction for risk, to models based on calibration and treatment benefit, and now to models based on machine learning.

Selecting the optimal mode of revascularization for individual patients with complex coronary artery disease (CAD) remains challenging. Analysis of the risk benefit ratio of alternative strategies of revascularization is complex. It includes consideration of multiple issues and analysis includes interaction between these multiple issues (Table 1). Although study of the individual as well as the totality of these issues has been a goal of modern cardiovascular disease (CVD) therapy, it has been extremely difficult. Many questions remain and answers have evolved over the course of time. The SYNTAX (Synergy Between Percutaneous Coronary Intervention With TAXUS and Coronary Artery Bypass Surgery) trial by virtue of its design and now extended follow-up has provided unique data and many answers. It is the goal of this article to evaluate the strategies of revascularization (Table 1) in as much of the totality of factors as is possible, to identify these areas that form the basis of individual-based care recommendations and are the basis of the art and science of optimizing care.

**Table 1:** Factors Affecting the Strategies of Revascularization (CABG vs PCI)

Clinical presentation
Extent, location, and severity of disease
Specific anatomic characteristics: Bifurcation disease, lesion calcification, total occlusion
Specific baseline clinical characteristics and comorbidities both individually as well as assessment of the totality
Specific revascularization strategy
CABG: arterial vs venous grafts and mixed patterns
Completeness of revascularization
PCI: number and specific types of stents
Stage procedures
Completeness of revascularization
Residual disease
Preprocedural and postprocedural adjunctive therapy
Length of follow-up
Patient expectations and timeline of care patterns

CABG = coronary artery bypass grafting; PCI = percutaneous coronary intervention.

Before the SYNTAX trial, published randomized trials of revascularization in patients with 2- and 3-vessel CAD only enrolled 2% to 12% of those patients who were screened owing to stringent patient selection and broad exclusion criteria. This phenomenon was described by Frederic Mohr as “cherry picking,”^1^ and he deserves great credit for endorsing the all-comers concept in the SYNTAX trial, with the added option to enroll in nested registries, patients who interventional cardiologists did not want to treat because of the complexity of their anatomy (percutaneous coronary intervention [PCI] registry) and patients who surgeons did not want to operate on because of the prohibitive risks related to their comorbidities (coronary artery bypass graft [CABG] registry).

The SYNTAX trial was a landmark trial conducted in a real-world setting wherein the Heart Team (composed of interventional cardiologists and cardiothoracic surgeons supported by the study coordinator) was used to randomize patients equally eligible for CABG and PCI.^2^ During the trial design, the anatomic SYNTAX score (SXscore) was developed as a screening tool to force surgeons and interventional cardiologists to semi-quantitatively analyze the angiograms to establish the true extent and complexity of the coronary vasculature in terms of the number of lesions, their location, and complexity.^3^ The evolution, extensive application, and integration of the SXscore into contemporary practice and guideline recommendations has been remarkable.^4^ The 1-year study results of this SYNTAX were published in 2009.^2^

## Section 1: The Design and Main Findings of the SYNTAX Trial

Before the SYNTAX trial, there were few adequately powered randomized trials of PCI in patients with complex CAD,^5^ with only a fraction of the patients treated in real-life clinical settings fulfilling the multiple inclusion and exclusion criteria, precluding a real-world assessment. Five-year follow-up of the ARTS (Arterial Revascularization Therapies Study) demonstrated a similar mortality rate for stenting vs CABG in patients with 2- and 3-vessel CAD.^6^ Notably, however, interpretation of this and other randomized controlled trials (RCTs) conducted before SYNTAX are severely limited by the absence of any quantitative assessment of the severity of CAD, and the subsequent inability to compare outcomes based on the extent and complexity of disease.

**Figure 1:**
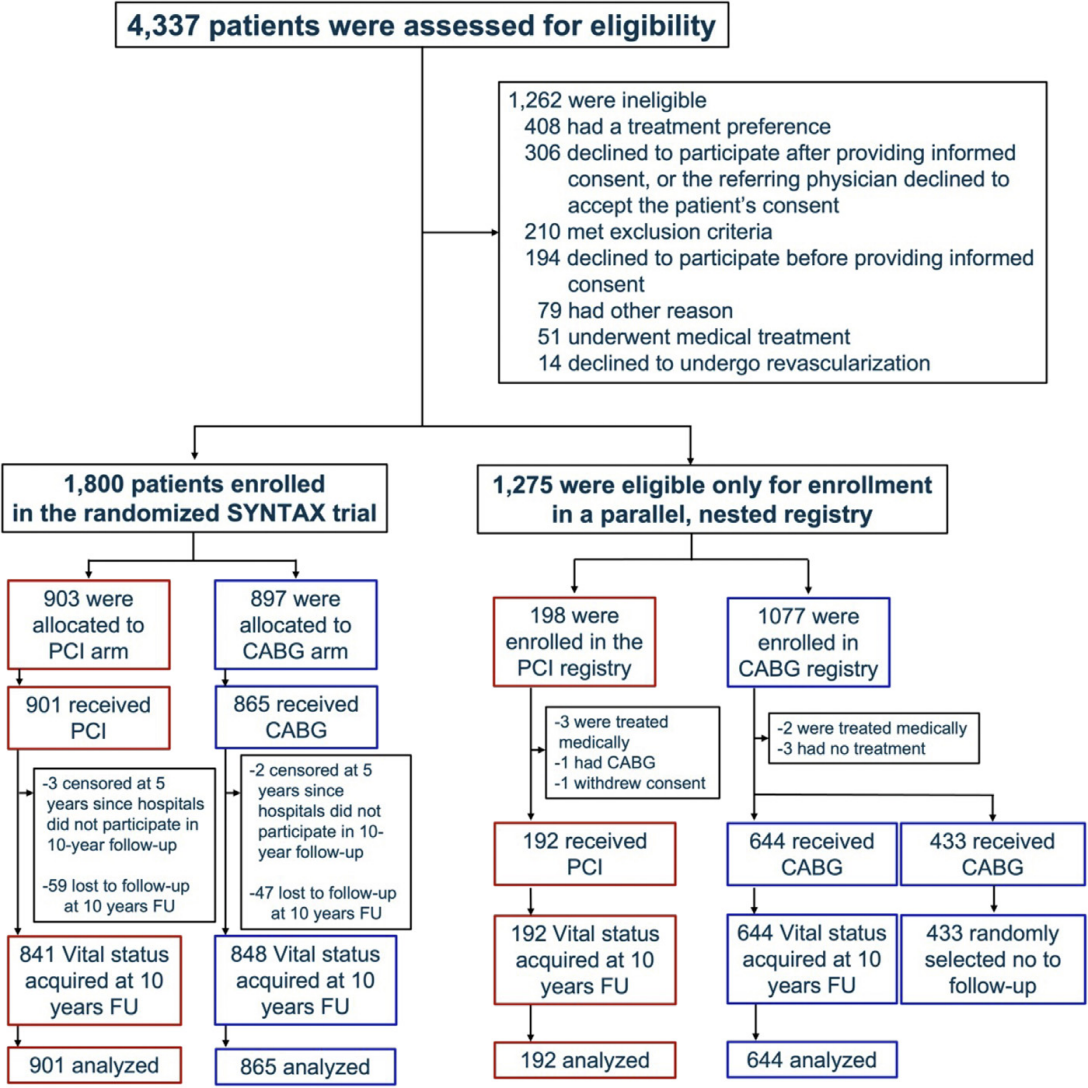
SYNTAX Study Design Flow Chart Consecutive patients with 3VD or LM disease were screened through the local Heart Team conference. LM = left main; SYNTAX = Synergy between PCI with Taxus and Cardiac Surgery; 3VD = 3-vessel disease

The SYNTAX trial assessed the optimal revascularization strategy for patients with de novo 3-vessel disease (3VD) or left main (LM) CAD (either isolated or in combination with 1, 2, or 3VD) by randomizing patients to either PCI with a polymer-based, paclitaxel-eluting TAXUS Express^2^ (Boston Scientific) stent or CABG. The study had an “all-comers” design with consecutive enrollment of all eligible patients with 3VD or LMCAD.^7^ Between March 2005 and April 2007, 4,337 patients were screened (Figure 1), and of these 1,800 with LMCAD and/or 3VD were randomized to CABG (n = 897) or PCI (n = 903) at 1 of 23 sites in the United States (n = 245) and 62 sites in Europe (n = 1,555). Almost 30% of screened patients were found to be suitable for only 1 treatment and entered the CABG (n = 1,077) or PCI (n = 198) nested registries, and 9.4% of patients were not willing to participate or had a preferred treatment.

At 1-year follow-up, CABG as compared with PCI resulted in lower rates of the combined endpoint of major adverse cardiac or cerebrovascular events (MACCE) (CABG 12.4% vs PCI 17.8%; *P* = 0.002) (Table 2), with the difference largely driven by significantly higher rates of repeat revascularization with PCI (13.5% vs 5.9%, *P* < 0.001).^2^ Results in the overall population at 5 years were consistent with the PCI group having significantly higher rates of MACCE (37.3% vs 26.9%; *P* < 0.0001) (Table 2),^8^ with the differences driven by the higher risk of myocardial infarction (MI) (9.7% vs 3.8%; *P* < 0.0001) and repeat revascularization (25.9% vs 13.7%; *P* < 0.0001) with PCI, whereas no significant between-treatment differences were seen for all-cause death (CABG 11.4% vs PCI 13.9%; *P* = 0.10) or stroke (CABG 3.7% vs PCI 2.4%; *P* = 0.09). In patients with low SXscore (≤22), MACCE was similar between PCI and CABG in the overall population (CABG 28.6% vs PCI 32.1%; *P* = 0.43) and when divided by the presence of 3VD (CABG 26.8% vs PCI 33.3%; *P* = 0.21) or LMCAD (CABG 31.0% vs PCI 36.9%; *P* = 0.12). In contrast, among patients with intermediate (23-32) or high (≥33) SXscores, MACCE was significantly higher with PCI (intermediate score CABG 25.8% vs PCI 36.0%; *P* = 0.008; and high score 26.8% vs 44.0%; *P* < 0.0001).^8^ Because there was a hierarchical statistical approach implying global noninferiority as a prerequisite for subgroup analysis, specific noninferiority in subgroups (eg, LMCAD vs 3VD) could not be claimed. The EXCEL (Evaluation of XIENCE versus Coronary Artery Bypass Graft Surgery for Effectiveness of Left Main Revascularization) study was subsequently designed to test this hypothesis.

**Table 2:** MACCE at 1 and 5 Years and All-Cause Mortality at 10-Year Follow-Up in the SYNTAX Trial

	Combined Endpoint of MACCE at 1 and 5 Years and All-Cause Mortality at 10 Years		
	CABG	PCI	*P* Value
1-y follow-up	12.4	17.8	0.002
5-y follow-up	26.9	37.3	<0.0001
10-y follow-up	28	24	0.066

Values are %.

CABG = coronary artery bypass grafting; MACCE = major adverse cardiac or cerebrovascular event(s); PCI = Percutaneous Coronary Intervention; SYNTAX = Synergy Between percutaneous coronary intervention with Taxus and coronary artery bypass surgery.

**Table 3:** Clinical Variables and Adjusted HRs

Risk Factors	Adjusted HR (95% CI)
Patients on OMT vs those on ≤2 types of medications at 5 y	0.47 (0.292-0.757)
Higher PCS (10-point increase in PCS) vs Lower PCS	0.84 (0.73-0.97)
Higher MCS (10-point increase in MCS) vs Lower MCS	0.85 (0.76-0.95)
Female vs male	1.02 (0.76-1.36)
Major infections within 60 d after index procedure (5- to 10-year mortality) vs no major infections	1.10 (0.62-1.96)
Prior CVD vs no prior CVD	1.35 (1.04-1.73)
CRP ≥2 mg/L vs CRP <2 mg/L	1.35 (1.01-1.82)
Established CVD vs no established CVD	1.40 (1.08-1.80)
CrCl ≥60 mL/min vs CrCl <60 mL/min	1.46 (1.07-2.00)
HbA1c ≥6% vs HbA1c <6% (42 mmol/mol)	1.51 (1.16-1.95)
LVEF (≤40% vs >40%)	1.562 (0.988-2.467)
DM (pharmacologically treated) vs no DM	
Overall cohort	1.58 (1.27-1.95)
PCI arm	1.54 (1.15-2.06)
CABG arm	1.65 (1.19-2.28)
No statin therapy vs statin therapy	1.68 (1.26-2.25)
COPD vs no COPD	2.03 (1.56-2.64)
Current smokers vs never smokers	2.29 (1.60-3.27)
Insulin-treated DM vs no DM	
Overall cohort	2.06 (1.57-2.70)
PCI arm	2.31 (1.55-3.43)
CABG arm	2.00 (1.35-2.96)
BMI/WC	
Low BMI/Low WC, Low BMI/High WC, High BMI/High WC vs High BMI/Low WC group	
Low BMI/Low WC	1.65 (1.09-2.51)
Low BMI/High WC	2.74 (1.12-6.69)
High BMI/High WC	1.59 (1.11-2.27)

BMI = body mass index; COPD = chronic obstructive pulmonary disease; CABG = coronary artery bypass graft surgery; CrCl = creatinine clearance; CRP = C-reactive protein; CVD = cardiovascular disease; DM = diabetes mellitus; LVEF = left ventricular ejection fraction; MCS = mental component score; OMT = optimal medical therapy; PCI = percutaneous coronary intervention; PCS = physical component score; WC = waist circumference.

**Table 4:** Procedural Variables and Adjusted HRs

Risk Factors	Adjusted HR (95% CI)
Single or multiple CABG vs PCI	
MAG	0.66 (0.49-0.89)
SAG	0.83 (0.67-1.03)
MAG vs SAG	0.74 (0.55-0.98)
On-pump CABG vs PCI	0.79 (0.65-0.96)
Proximal LAD lesion vs no proximal LAD lesion	0.95 (0.72-1.26)
Off-pump CABG vs PCI	0.98 (0.69-1.40)
TO vs non-TO	1.018 (0.810-1.280)
Total stent length as a continuous variable	1.05 (1.01-1.09) per 10-mm increase
Residual angina vs no residual angina	1.11 (0.83-1.47)
TO recanalization/revascularization vs non-TO recanalization/revascularization	
PCI arm	1.041 (0.645-1.681)
CABG arm	1.311 (0.746-2.303)
≥1 Heavily calcified lesion vs no calcified lesion	1.36 (1.09-1.69)
Proximal LAD lesion (PCI vs CABG)	1.39 (0.99-1.95)
Nonfatal PMAEs (mortality between 30 d and 10 y) vs no nonfatal PMAEs	1.46 (1.16-1.82)
PCI and >1 Bifurcation (2 stents vs 1 stent)	1.51 (1.06-2.14)
>1 Bifurcation lesion vs no bifurcation	
PCI group	1.55 (1.12-2.14)
CABG group	0.81 (0.59-1.12)
SPCI vs no SPCI	1.69 (1.23-2.32)
PCI with small stents (<3 mm) vs CABG and PCI with small stents (<3 mm) vs PCI with large stents (≥3 mm)	1.66 (1.23-2.26) when compared with CABG 1.74 (1.19-2.53) when compared with large stents PCI
≥2 nonfatal PMAEs vs no PMAEs	1.78 (1.11-2.86)
Extensive stenting PCI (>100 mm) vs CABG and extensive stenting PCI vs not-extensive stenting PCI	1.97 (1.41-2.74) when compared with CABG 1.94 (1.36-2.77) when compared with not- extensive stenting PCI
Periprocedural MI vs no periprocedural MI	
SYNTAX definition	3.26 (1.92-5.51)
4th UDMI	3.00 (1.74-5.16)
SCAI/EXCEL definition	1.32 (0.90-1.92)
PCI with rSS >8 vs PCI with complete revascularization (rSS of 0)	3.40 (2.13-5.43)

CABG = coronary artery bypass graft surgery; EXCEL = Evaluation of XIENCE versus Coronary Artery Bypass Graft Surgery for Effectiveness of Left Main Revascularization; LAD = left anterior descending artery; MAG = multiple arterial grafts; PCI = percutaneous coronary intervention; PMAEs = periprocedural major adverse event(s); rSS = residual SYNTAX score; SPCI = staged percutaneous coronary intervention; SAG = single arterial graft; SCAI = Society for Cardiovascular Angiography and Interventions; SYNTAX = Synergy Between percutaneous coronary intervention with Taxus and coronary artery bypass surgery; TO = total occlusion; 4th UDMI = Fourth Universal Definition of Myocardial Infarction.

SYNTAXES (SYNTAX Extended Survival) was an investigator-driven, retrospective follow-up study designed to compare long-term survival data (10-year follow-up) of patients previously enrolled in the SYNTAX trial. At 10 years in the overall cohort, no significant difference existed in all-cause death between PCI using first-generation drug-eluting stents and CABG (PCI 27.46% vs CABG 23.63%; HR: 1.19; 95% CI: 0.99-1.43; *P* = 0.066) (Table 2); however morality among patients with 3VD was higher following PCI compared with CABG (PCI 28% vs CABG 21%, HR: 1.42; 95% CI: 1.11-1.81), and similar among those with LMCAD (PCI 27% vs CABG 28%, HR: 0.92; 95% CI: 0.69-1.22; *P*_interaction_ = 0.023). It was concluded therefore that CABG provided a significant survival benefit in patients with 3VD, but not in those with LMCAD.^9^

There is abundant literature on the use of composite primary endpoints and their subcomponents in trials comparing PCI with CABG for myocardial revascularization. ARC II definition for patient-oriented composite endpoints include by hierarchical order all-cause mortality, any stroke, any MI, and any revascularization. For instance, whether a stroke equates to an MI or alternatively amounts to an MI plus 1 target vessel revascularization episode has been a longstanding source of debate. Undoubtedly, composite primary endpoints are practical but also suboptimal. Their post hoc splitting and pooling also can lead to methodological shortcomings.^10^

Individual endpoint-related questions that are relevant to recent RCTs comparing PCI with CABG include the following: Does target vessel revascularization constitute a benign outcome, despite the paucity of dedicated literature examining its late effects? Should periprocedural MI, arbitrarily defined by enzyme release thresholds that vary from 1 trial to another using biochemical assays that also fluctuate from 1 laboratory to another, represent an important hypothesized clinical outcome difference between PCI and CABG?^10^

On these issues, the latest 2 trials, NOBLE (Nordic–Baltic–British left main revascularization trial ) and EXCEL, took opposite approaches. NOBLE, like previous trials, included target vessel revascularization as part of its composite primary endpoint, whereas EXCEL did not. Furthermore, NOBLE did not consider periprocedural MI to be an important and comparable source of clinical difference and did not include it in its composite primary endpoint. What happened in this regard in the EXCEL trial is noteworthy.^10^ In the future trials comparing PCI and CABG for revascularization in 3VD and LM disease, the optimal primary endpoint would be still a composite of all-cause death, MI, stroke, and repeat revascularization, although whether periprocedural MI would be included as a subcomponent of MI is debatable.

## Section 2: Prespecified Analyses From the SYNTAX Trial

Considering the size of the SYNTAX study and its all-comers design, with minimal exclusion, the cohort offers ample opportunity to try to identify a population that has most to gain from either CABG or PCI (Central Illustration). In the following is a summary of the results from prespecified analyses from SYNTAX and SYNTAXES.

### Sex

**Figure 2:**
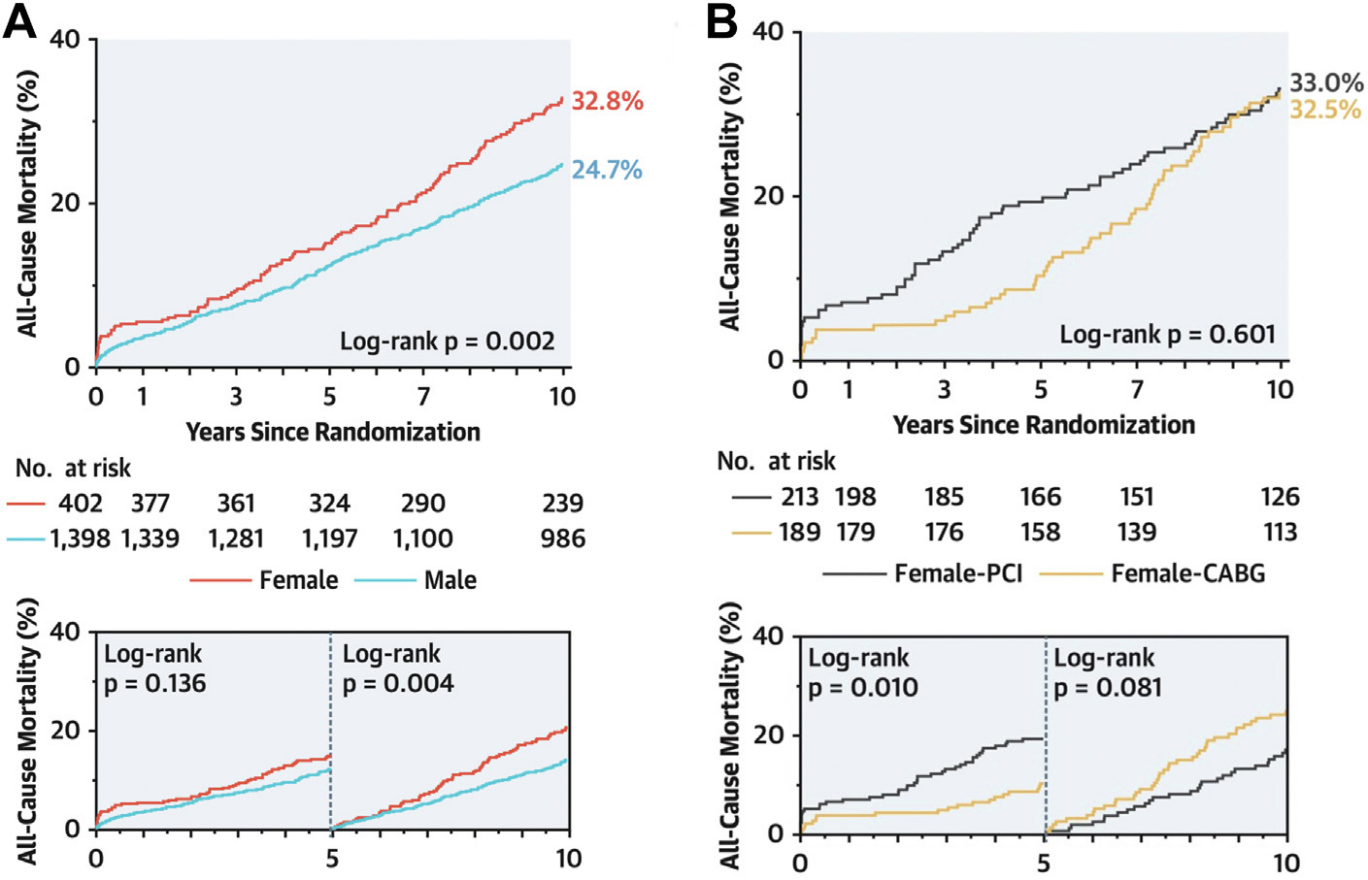
All-Cause Mortality at 10 Years According to Sex **(A)** Kaplan-Meier curves for all-cause mortality at 10 years according to sex. **(B)** Kaplan-Meier curves for all-cause mortality at 10 years in females according to treatment strategy. Modified with permission from Hara et al.^11^

At 5 years, morality following revascularization with PCI vs CABG differed significantly between females and males and based on this significant sex-treatment interaction, female sex was incorporated into the SYNTAX score II (SSII). At 10 years, mortality was still higher with females compared with males (log-rank *P* = 0.002); however, female sex was no longer an independent predictor of mortality (*P* = 0.915) (Figure 2A). Overall, 10-year mortality tended to be lower after CABG than after PCI, with a similar relative treatment effect between the sexes (*P*_interaction_=0.952),^11^ such that female sex was no longer retained in the redeveloped and calibrated SYNTAX Score II 2020 (SSII-2020) risk model.^12^ Of note, the significant mortality benefit with CABG observed in females at 5 years disappeared at 10 years (Figure 2B, Table 3).

### Diabetes

**Figure 3:**
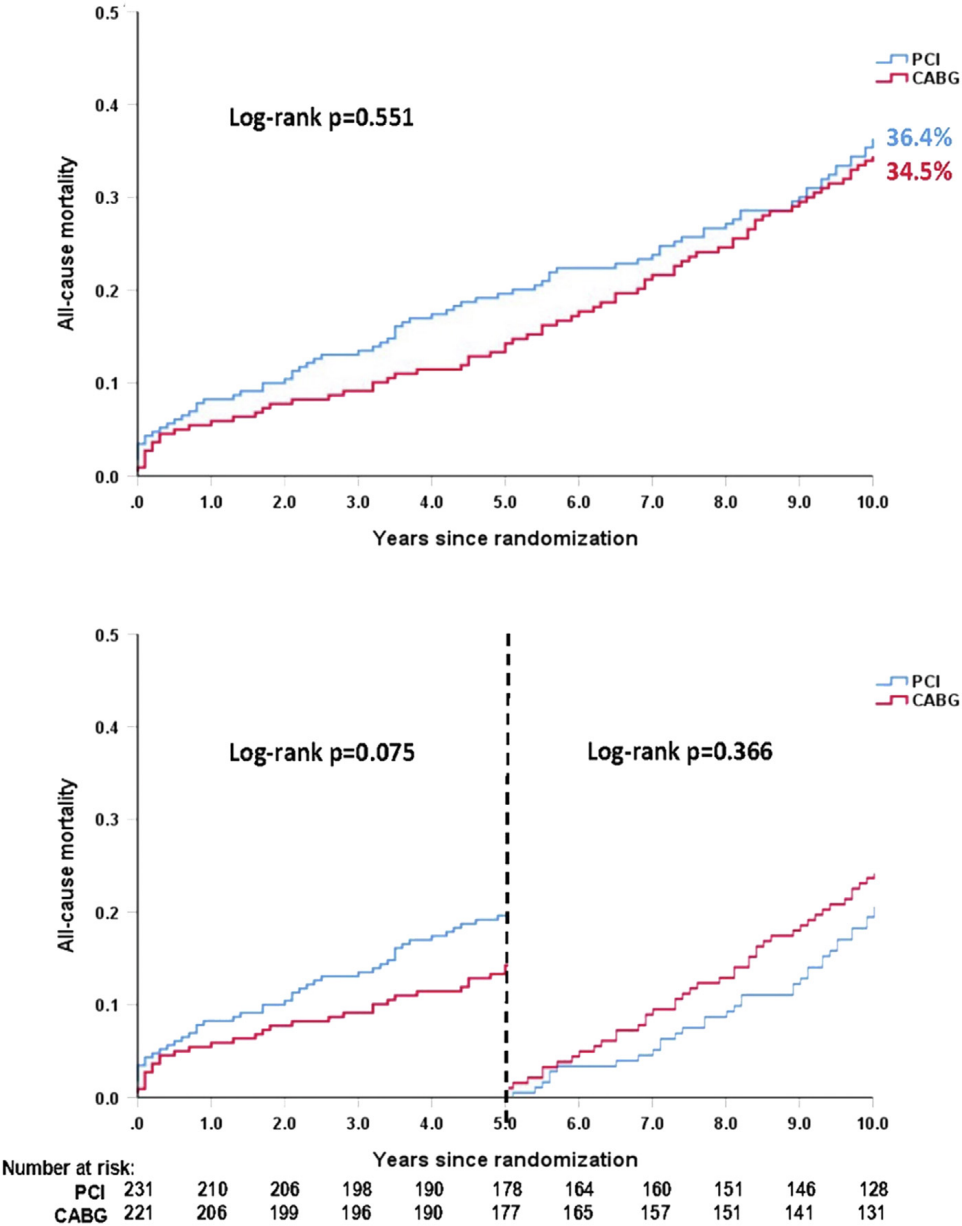
All-Cause Mortality at 10 Years in Patients With Diabetes Mellitus Kaplan-Meier curves for all-cause mortality at 10 years according to treatment strategies in patients with diabetes mellitus. Reproduced with permission from Wang et al.^13^

Compared with patients without diabetes mellitus (DM), pharmacologically treated DM was an independent predictor of all-cause death at 10 years in the overall cohort (*P* < 0.001), and among those treated with PCI (*P* = 0.003) and CABG (*P* = 0.003). Patients in the SYNTAX study with DM had significantly higher rates of MACCE at 5 years when treated with PCI vs CABG, which was mainly driven by a higher rate of repeat revascularization. Furthermore, although the risk of mortality among diabetic individuals was numerically higher with PCI compared with CABG at 5 years (*P* = 0.075) the opposite was seen between 5 and 10 years (*P* = 0.366) (Figure 3). Among insulin-treated patients, all-cause death at 10 years was numerically higher with PCI (47.9% vs 39.6%; *P* = 0.227). Nevertheless, irrespective of diabetic status, there was no significant treatment effect of PCI vs CABG on 10-year mortality (DM, *P* = 0.551; no DM, *P* = 0.076; *P*_interaction_ = 0.856).

Diabetic patients with 3VD had comparable mortality with PCI and CABG at 10 years (*P* = 0.289); however, landmark analysis showed that mortality was significantly higher with PCI at 5 years (*P* = 0.020), whereas it was numerically higher with CABG between 5 and 10 years (*P* = 0.295). In nondiabetic patients with 3VD, the risk of mortality was significantly higher with PCI at 5 (*P* = 0.007) and 10 years (*P* = 0.001). In patients with LMCAD, there was no significant treatment effect of PCI compared with CABG on morality at 10 years among patients with (*P* = 0.781) or without (*P* = 0.455) DM^13^; however, Boden et al^14^ raised several concerns regarding these conclusions in their accompanying editorial (Table 3).

### Elderly

**Figure 4:**
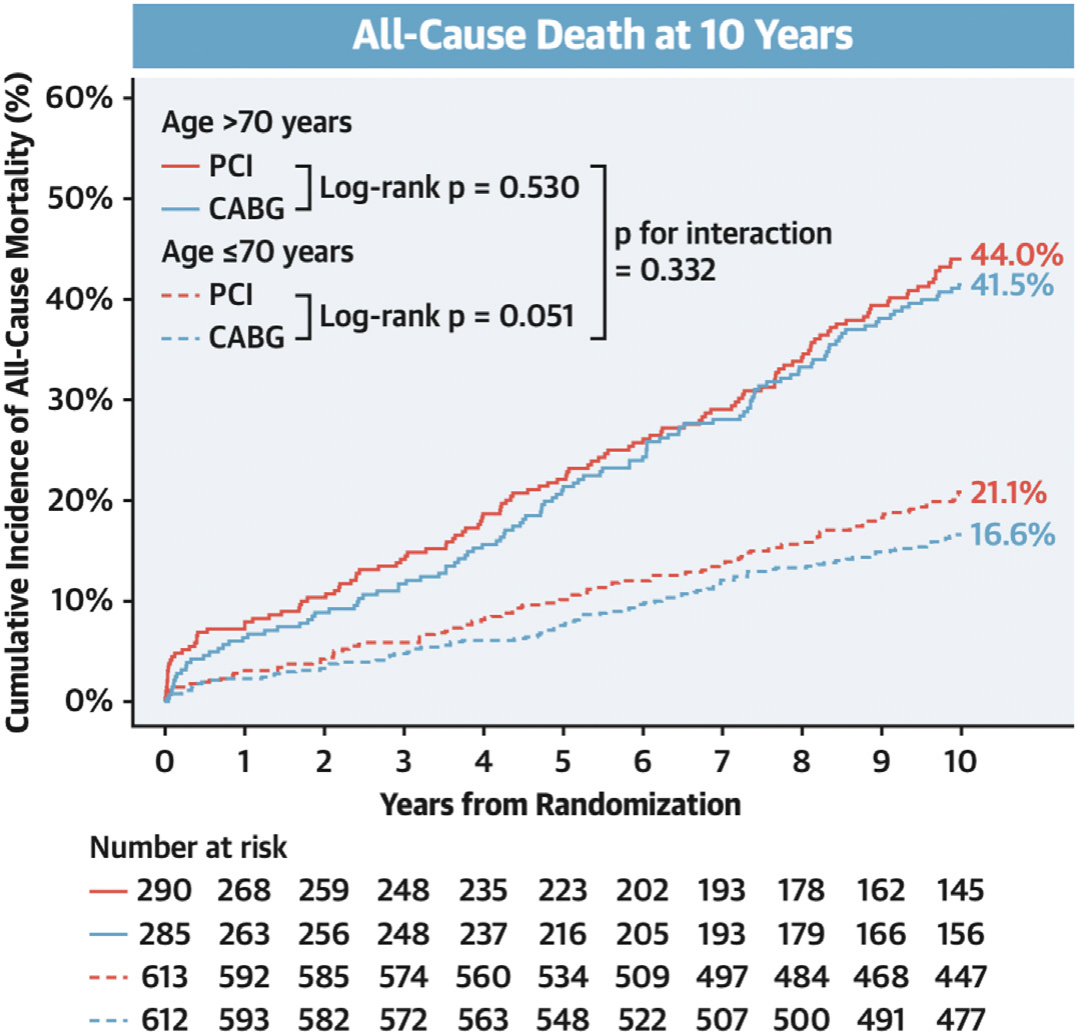
All-Cause Mortality at 10 Years in Elderly vs Nonelderly Individuals Kaplan-Meier curves show the cumulative incidence of all-cause death at 10 years in coronary artery bypass graft (CABG) **(blue curves)** or percutaneous coronary intervention (PCI) **(red curves)** arms stratified by age older than or younger than 70 years. Reproduced with permission from Ono et al.^15^

Approximately a third of the study cohort were elderly (>70 years) and 10-year mortality did not differ significantly following PCI or CABG among elderly and nonelderly patients (*P*_interaction_ = 0.332) (Figure 4). Elderly patients with 3VD and/or LMCAD had comparable 10-year mortality, life expectancy, 5-year MACCE, and 5-year quality of life status irrespective of the modality of revascularization. Of note, in elderly patients undergoing CABG fewer arterial conduits and more venous conduits were used compared with the nonelderly group.^15^

### Smoking

**Figure 5:**
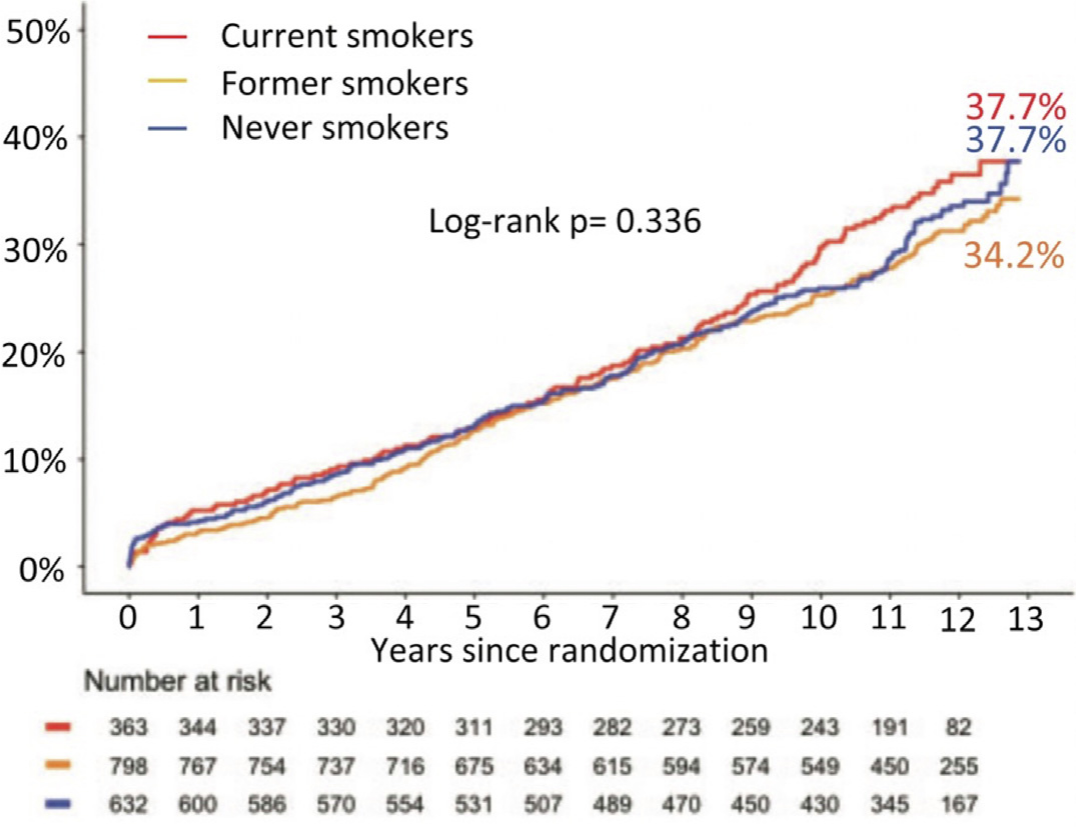
All-Cause Mortality at Maximum Follow-Up With Respect to Smoking Status Kaplan-Meier curves for all-cause mortality at maximum follow-up of 12.9 years in current **(red)**, former **(orange)**, and never **(blue)** smokers among the overall cohort. Reproduced with permission from Takahashi et al.^18^

Current smokers, who were on average 10 years younger than nonsmokers, made up approximately 20% of the study cohort, and had a significantly higher risk of 10-year all-cause mortality compared with those who had never smoked, whereas former smokers did not. This suggests that the “smokers paradox” does not exist in stable patients with complex CAD.^16^^,^^17^ Current smokers had a higher risk of all-cause mortality following PCI compared with CABG; however, no significant interaction was seen between revascularization strategy and smoking status (*P*_interaction_ = 0.910). In the CABG arm, the crude rates of all-cause mortality were similar irrespective of smoking status (Figure 5).^18^ However, in conjunction with data from FREEDOM, smoking status (current smoker) at the time of randomization was retained in the SSII-2020 as an independent predictor of all-cause mortality (Table 3).

## Section 3: Post Hoc Analyses

### Clinical conditions. Impact of left ventricular ejection fraction

**Figure 6:**
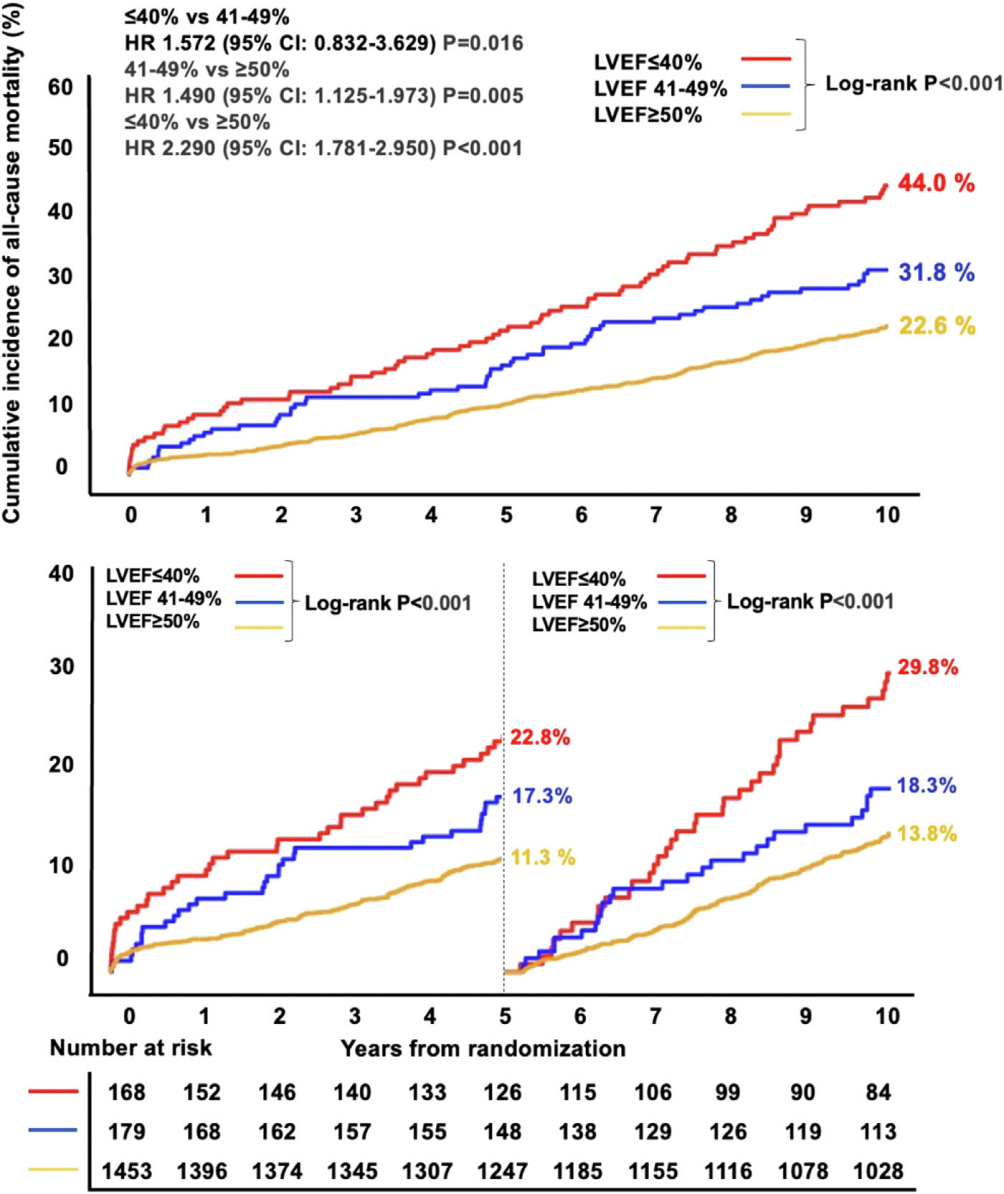
All-Cause Mortality at 10 Years With Respect to LVEF Kaplan-Meier curves for all-cause mortality at 10 years for each left ventricular ejection fraction (LVEF) subgroup. Landmark analysis shows a continuous and significant divergence in the cumulative incidence of mortality beyond 5 years.

The impact of reduced left ventricular ejection fraction (LVEF) on very long-term prognosis following PCI or CABG remains to be established. Current guidelines recommend CABG as the standard treatment for revascularization in patients with multivessel disease and LVEF ≤35% (Central Illustration). Patients with reduced ejection fraction (rEF) had a higher prevalence of 3VD compared to patients with mid-range ejection fraction (mrEF) and preserved ejection fraction (pEF) (70.2% vs 63.1% vs 59.5%; *P* = 0.020). At 10 years, all-cause mortality was 44.0% vs 31.8% vs 22.6%, in patients with rEF, mrEF, and pEF, respectively (*P* < 0.001), with a significant difference first emerging at 5-year follow-up (Figure 6). Overall, patients with an LVEF <50% had a poorer prognosis than those with pEF. Ten-year mortality in patients with rEF was higher with PCI than CABG, but this difference was not statistically significant (52.9% vs 39.6%; *P* = 0.054) presumably due to the small sample size (n = 168; PCI group: n = 77; CABG group: n = 91). The significant interaction for all-cause mortality between LVEF subgroups and revascularization modality at 5-year follow-up was no longer seen at 10 years (Figure 6, Table 3).

**Central Illustration:**
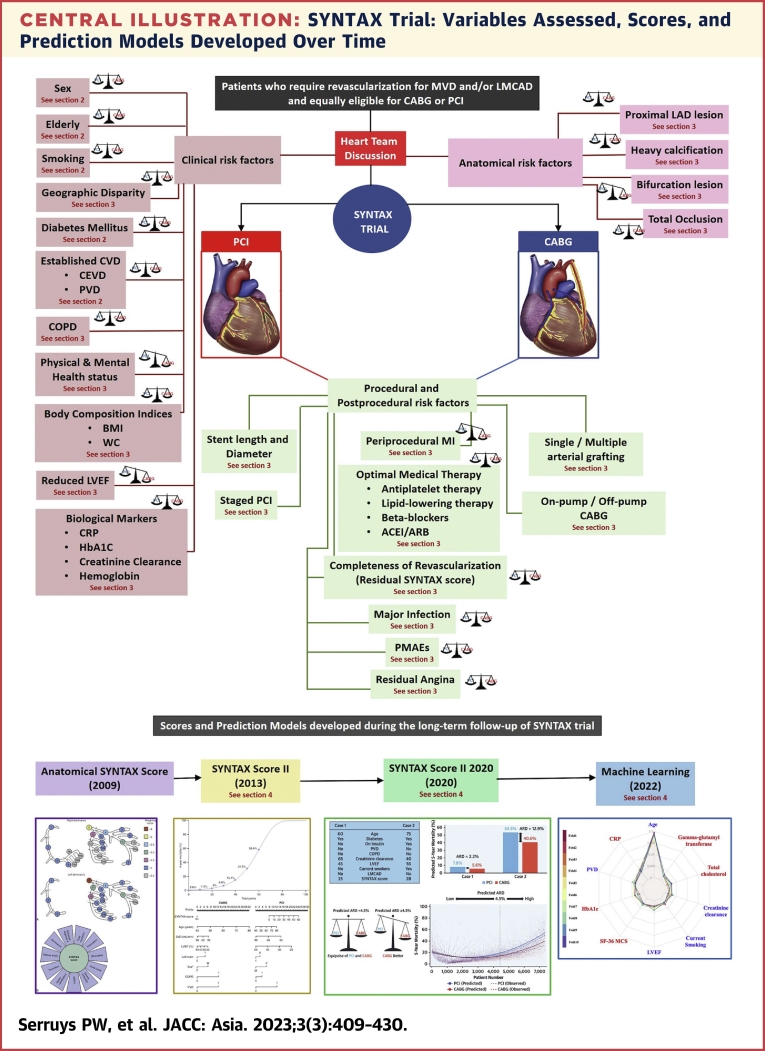
SYNTAX Trial: Variables Assessed, Scores, and Prediction Models Developed Over Time SYNTAX trial used the Heart Team approach for the first time to randomize patients equally eligible for both CABG and PCI. Several clinical, procedural, and postprocedural variables and their impact on 10-year mortality have been assessed since then. Various scoring systems namely anatomical SYNTAX score and SYNTAX score II, and prediction models (SYNTAX score II) were developed during the course of the trial to individualize risk among patients undergoing revascularization. Certainly, we have seen the evolution of risk factor assessment from conventional multivariable models to models based on the interaction for risk, to models based on calibration and treatment benefit, and now to models based on machine learning. BMI = body mass index; CABG = coronary artery bypass grafting; CVD = cardiovascular disease; CEVD = cerebrovascular disease; COPD = chronic obstructive pulmonary disease; LAD = left anterior descending coronary artery; LVEF = left ventricular ejection fraction; LMCAD = left main coronary artery disease; MI = myocardial infarction; MVD = multivessel disease; PCI = percutaneous coronary intervention; PMAE = periprocedural major adverse event(s); PVD = peripheral vascular disease; WC = waist circumference; SYNTAX = Synergy Between percutaneous coronary intervention with Taxus and coronary artery bypass surgery.

### Body composition indices

**Figure 7:**
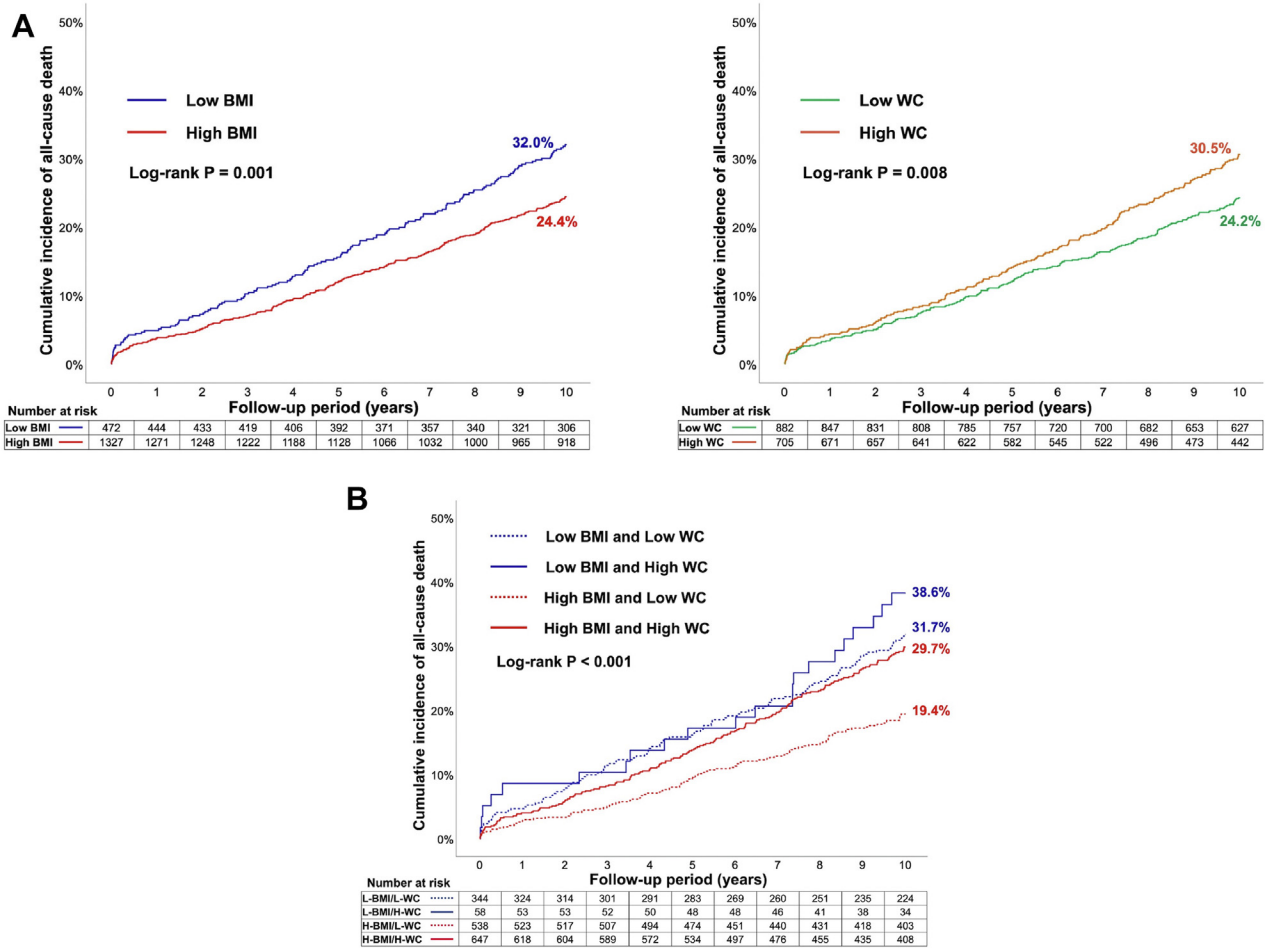
All-Cause Mortality at 10 Years With Respect to Body Composition Indices **(A)** Kaplan-Meier curves for 10-year all-cause death in the body mass index (BMI) or waist circumference (WC) groups. Kaplan-Meier curves in patients with low or high BMI **(left)**, or those with low or high WC **(right)**. **(B)** Kaplan-Meier curves for all-cause mortality at 10 years in patients stratified by both BMI and WC. Cumulative incidence of all-cause death up to 10 years in patients with low BMI/low WC, low BMI/high WC, high BMI/low WC, and high BMI/high WC. Reproduced with permission from Ono et al.^19^

Seventy-four percent of the study cohort had a body mass index (BMI) ≥25 kg/m^2^ and 44.4% had a waist circumference (WC) >102 cm for men or >88 cm for women. When stratified by both BMI and WC, crude 10-year mortality risk was significantly higher in patients with low BMI/low WC (adjusted HR [aHR]: –1.65), low BMI/high WC (aHR: – 2.74) or high BMI/high WC (aHR: – 1.59) compared with those with high BMI/low WC (Figure 7). After adjustment, there was no significant difference in the incidence of all-cause death at 10 years between high vs low BMI groups. Patients with high WC had a significantly higher crude rate of all-cause death at 10 years compared with those with low WC; WC was an independent predictor of 10-year mortality (Figure 7, Table 3).^19^

### Preprocedural biological markers

**Figure 8:**
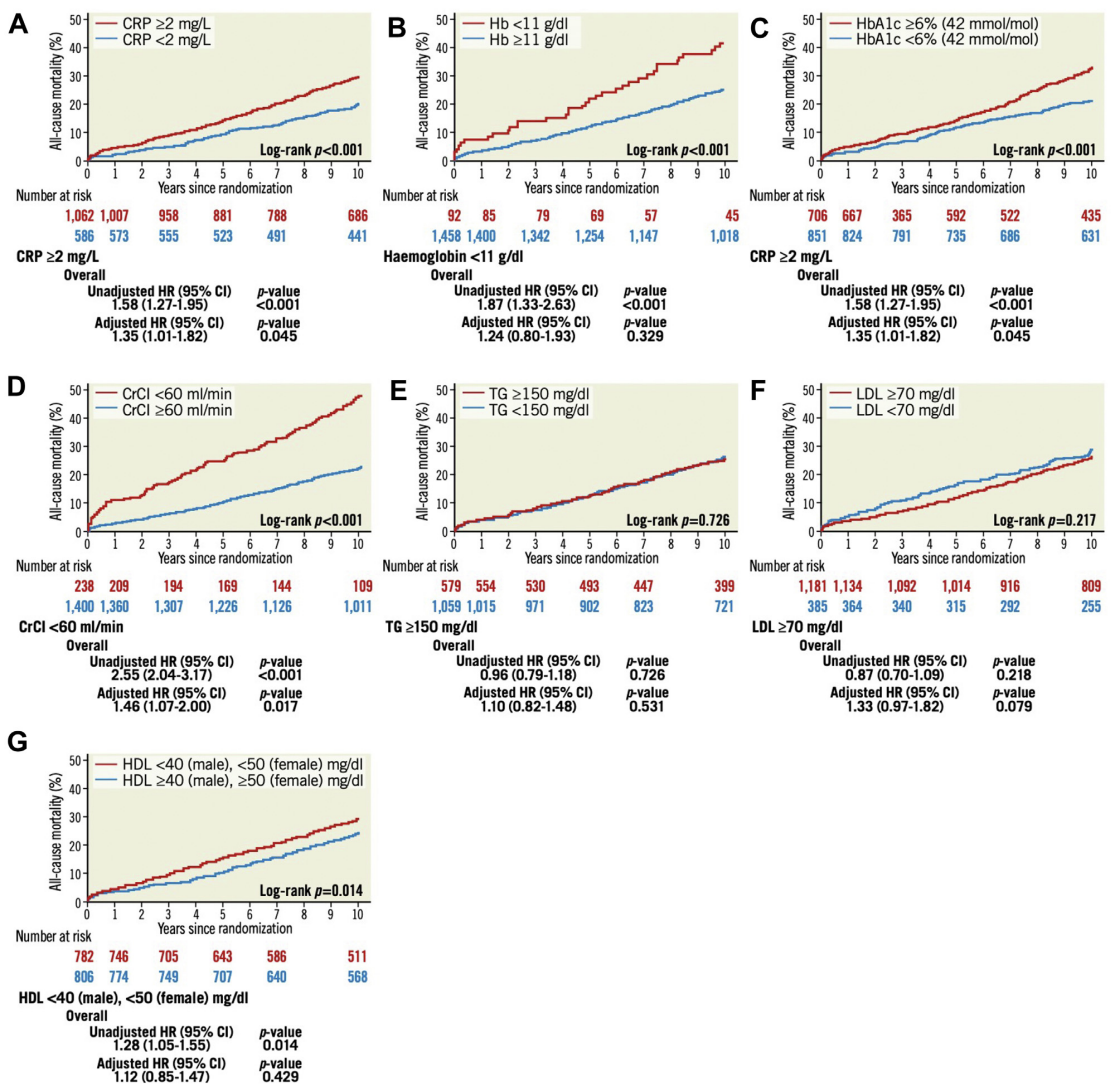
All-Cause Mortality at 10 Years With Respect to Biological Markers Mortality rates according to **(A)** C-reactive protein (CRP), **(B)** hemoglobin, **(C)** HbA1c, **(D)** creatinine clearance (CrCl), **(E)** triglycerides (TG), **(F)** low-density lipoprotein cholesterol (LDL-C), and **(G)** high-density lipoprotein cholesterol (HDL-C). HR with 95% CI of each categorical variable for 10-year mortality is shown in the **lower panel**. Reproduced with permission from Hara et al.^20^

C-reactive protein (CRP), HbA1c, and creatinine clearance (CrCl) with threshold values of ≥2 mg/L, ≥6% (42 mmol/mol) and <60 mL/min, respectively, were all associated with an increased risk of 10-year all-cause death (aHR: 1.35, 1.51, and 1.46) (Figure 8); however, there was no significant interaction on all-cause mortality between the biological markers and the type of revascularization. There was no significant correlation between preprocedural lipid markers (triglycerides, low-density lipoprotein cholesterol, and high-density lipoprotein cholesterol) and 10-year all-cause death; however, the non-use of statins was associated with worse prognosis (aHR: 1.68). The beneficial effect of statins was similar in patients with high (≥2 mg/L) or low (≤2 mg/L) preprocedural CRP. Notably, mortality rates at 10 years were consistently lower in patients treated with (between 21.4% and 23.2%), compared to without (between 28.8% and 50.9%) statins after discharge, irrespective of preprocedural lipid values. CRP (10 mg/L increase), hemoglobin (1 g/dL decrease) and HbA1c (1% [11 mmol/mol] increase) were significantly associated with 10-year all-cause death (aHR: 1.07, 1.08, and 1.15). Patients who did not have a history of diabetes at enrollment but had HbA1c ≥6% (42 mmol/mol) had higher mortality at 10 years (aHR: 1.38), especially after PCI (aHR: 1.82). In contrast, preprocedural HbA1c did not predict 10-year all-cause mortality in patients with medically treated diabetes at enrollment. Inclusion of biological markers into the SSII-2020 did not significantly improve predictivity.^20^ As is discussed later in contrast to the SSII-2020, machine learning (ML) algorithms identified CRP as one variable able to predict 10-year mortality (Table 3).

### Established CVD

**Figure 9:**
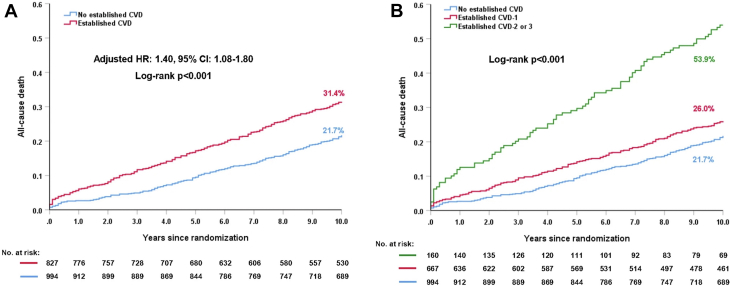
All-Cause Mortality at 10 Years According to the Established CVD **(A)** 10-year all-cause mortality in patients with established cardiovascular disease (CVD) vs those without. **(B)** 10-year all-cause mortality according to the extent of established CVD. Reproduced with permission from Wang et al.^21^

Nearly half of the study cohort had established CVD, defined as ≥1 prior MI, ≥1 prior cerebrovascular event, or established peripheral vascular disease (PVD), and these patients were older and had significantly higher rates of insulin-treated DM, chronic obstructive pulmonary disease (COPD), congestive cardiac failure, and unstable angina, compared with those without. In addition, they had higher EuroSCOREs, Parsonnet scores, anatomical SXscores, and lower LVEF. Compared with those without established CVD, patients with CVD had a significantly higher risk of 10-year all-cause death (*P* = 0.010), with this difference driven by those patients with CVD in ≥2 territories (Figure 9). The relative treatment effects of PCI vs CABG on 10-year all-cause death in patients with complex CAD were similar irrespective of the presence of established CVD (*P*_interaction_ = 0.986). Prior cerebrovascular disease and PVD were independent predictors of all-cause death at 10 years and maximum follow-up; however, prior MI was not (Table 3).^21^

### COPD

Approximately 9% of the study cohort had COPD, and regardless of revascularization strategy, these patients had a higher risk of 10-year all-cause death compared with those without (*P* < 0.001). The relative treatment effects of CABG vs PCI on 10-year all-cause death were not significantly different for patients with and without COPD, and although it was an independent predictor of 10-year all-cause death after CABG, it was not after PCI (Table 3).^22^

### Prior cerebrovascular disease

Fourteen percent of the study cohort had prior cerebrovascular disease (CEVD), defined as a prior stroke, transient ischemic attack, or carotid artery disease. Prior CEVD was an independent predictor of 10-year all-cause death (aHR: 1.35; *P* = 0.021). Patients with prior CEVD had a significantly higher risk of 10-year all-cause death (*P* < 0.001), with rates similar between patients receiving PCI or CABG, irrespective of the presence of a prior CEVD (*P*_interaction_ = 0.624) (Table 3).^23^

### Patient-reported preprocedural physical and mental health

**Figure 10:**
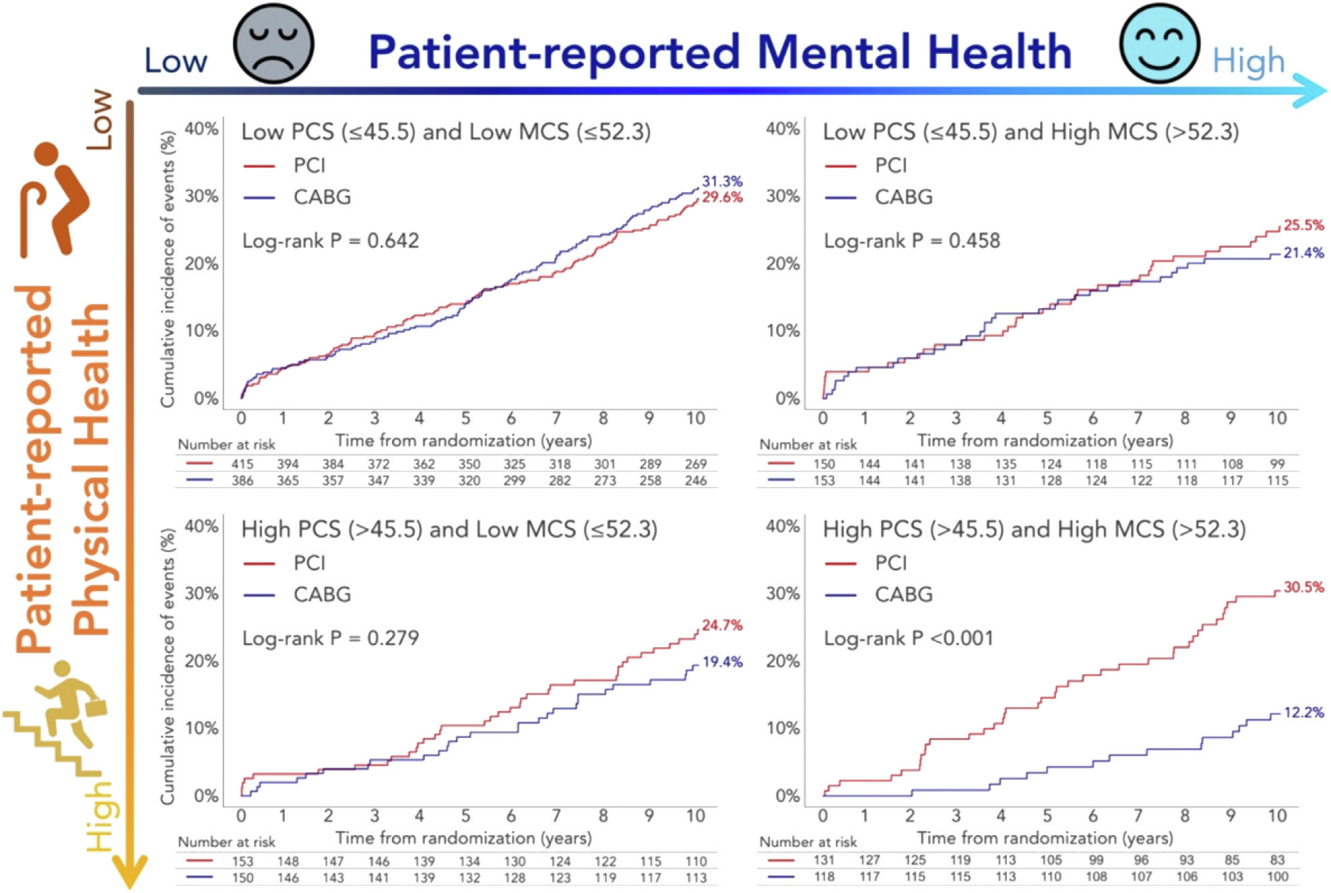
All-Cause Mortality at 10 Years Stratified by SF-36 PCS and MCS Patients were stratified by both 36-Item Short Form Health Survey (SF-36) physical component summary score (PCS) (≤45.5 or >45.5) and mental component summary score (MCS) (≤52.3 or >52.3). The survival benefit of CABG over PCI at 10 years was observed in patients with high PCS (>45.5) and high MCS (>52.3), whereas it was not observed in those with low PCS (≤45.5) or low MCS (≤52.3). Reproduced with permission from Ono et al.^24^ Abbreviations as in Figure 4.

Data from the preprocedural 36-item short form (SF-36) health survey were available in 92% of patients. Both higher physical component summary (PCS) and mental component summary (MCS) scores were independently associated with lower 10-year mortality; however, MCS was more independently relevant than PCS. A survival benefit with CABG over PCI was observed in the highest PCS and MCS tertiles with significant treatment-by-subgroup interactions (PCS *P*_interaction_ = 0.033, MCS *P*_interaction_ = 0.015). In patients with high PCS (>45.5) and MCS (>52.3), 10-year mortality was significantly higher with PCI compared with CABG (*P* = 0.001), whereas no significant treatment differences were seen among those with a low PCS (≤45.5) and/or a low MCS (≤52.3), resulting in a significant treatment-by-subgroup interaction (*P*_interaction_ = 0.002) (Figure 10, Table 3).^24^

### Geographic disparity

**Figure 11:**
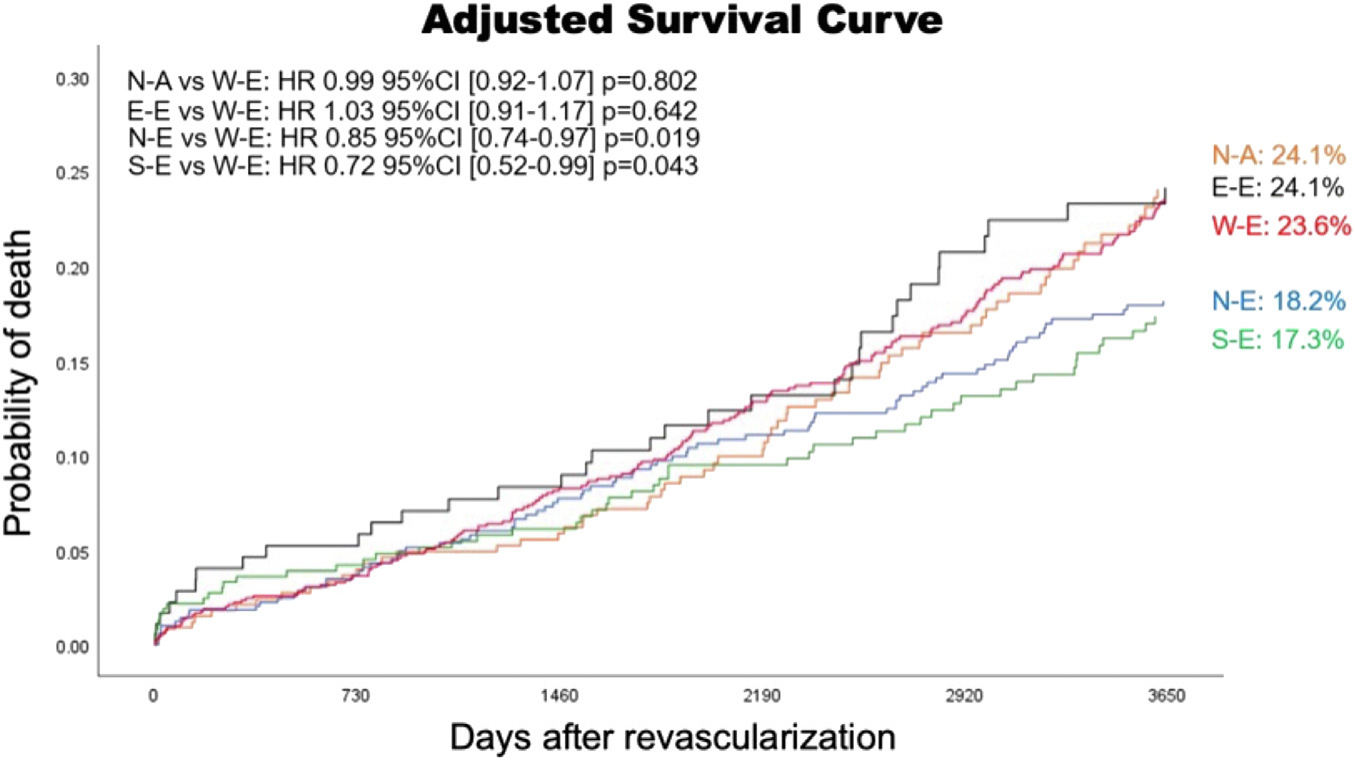
Adjusted Survival Curve of 5 Regions in the SYNTAXES Trial In this figure, HRs, CIs, and *P* values of 10-year mortality of each region against W-E using Cox regression analysis are presented on left upper side. Preprocedural characteristics used for adjustment of the survival are as follows; age, sex, medically treated diabetes, current smokers, peripheral vascular disease, chronic obstructive pulmonary disease, chronic kidney disease (defined as creatinine clearance <60 mL/min/1.73 m^2^), left ventricular ejection fraction (categorized <50% or not), disease type (left main coronary artery disease or 3-vessel disease), and anatomical SYNTAX score. Number of participants who could not be included in the analysis due to missing values in the whole population was 176 of 1,800 participants (9.8%). Reproduced with permission from Kageyama et al.^25^ E-E = Eastern Europe; N-A = North America; N-E = Northern Europe; S-E = Southern Europe; W-E = Western Europe.

**Figure 12:**
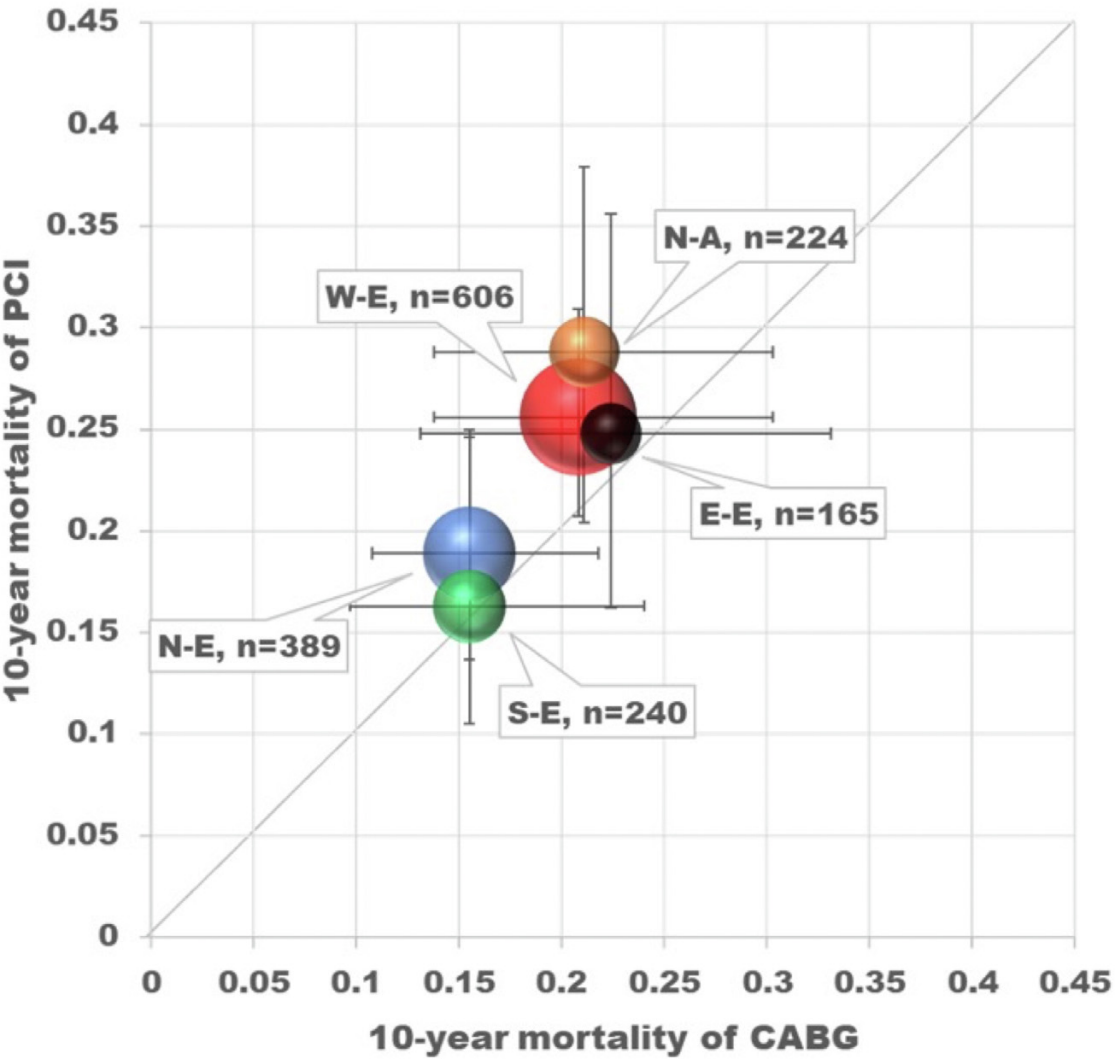
Relationship Between 10-Year Mortality Per Revascularization Strategy and Regions Bubble chart of relations between adjusted 10-year mortality per revascularization strategy and the region. (right side) showed forest plot presenting HR of PCI arm against CABG arm in the 5 regions. *P* for interaction between revascularization and region was 0.302. Reproduced with permission from Kageyama et al.^25^ Abbreviations as in Figures 4 and 11.

Global trials will always face issues due to the extensive heterogeneity in baseline characteristics, socioeconomics, genomic factors, techniques of revascularization, and quality of operators, as well as postprocedural medication, and lifestyle postrevascularization. Regional variations in morbidity and cardiovascular mortality also exist, which has been attributed to factors such as household income, medical insurance, provision and availability of health care services and equipment, density of doctors and hospitals, and other less well-identified factors such as diet and regional epigenetics. Rates of crude 10-year mortality were significantly lower in eastern (22.5%; *P* = 0.041), northern (21.9%; *P* = 0.003), and southern Europe (22.0%; *P* = 0.014) compared with western Europe (30.7%) and North America (31.6%) (Figures 11 and 12). The differences in 10-year mortality remained significantly lower with northern and southern Europe even after adjustment for confounding factors, including the SSII-2020. No statistically significant interactions were seen between geographic location and revascularization strategy. The rates, and trends of prescribing optimal medical therapy (OMT) over the first 5 years were very heterogeneous among the 5 regions, and low rates in North America, compared with Europe, may explain their sharp increase in mortality after 5 years, in addition to their high usage of venous conduits.^25^

### Procedural characteristics

See the Supplemental Appendix, Supplemental Figures 1 to 11, Table 4, and the Central Illustration.

### Postprocedural variables

See the Supplemental Appendix, Supplemental Figures 1 to 11, Table 4, and the Central Illustration.

## Section 4: Risk Balance Between PCI and CABG—Prediction Models

See the Central Illustration.

### Average treatment effect vs personalized decision making

RCTs are considered the gold standard for testing the efficacy of novel therapeutic interventions, and typically report the average treatment effect as a summary effect. The outcomes from treatment can vary between patients, and therefore basing treatment decisions for individual patients on the overall average treatment effect could be suboptimal.^26^ Thus individualized decision-making tools are essential to help select the optimal revascularization strategy in patients with complex CAD.

### SYNTAX score II

**Figure 13:**
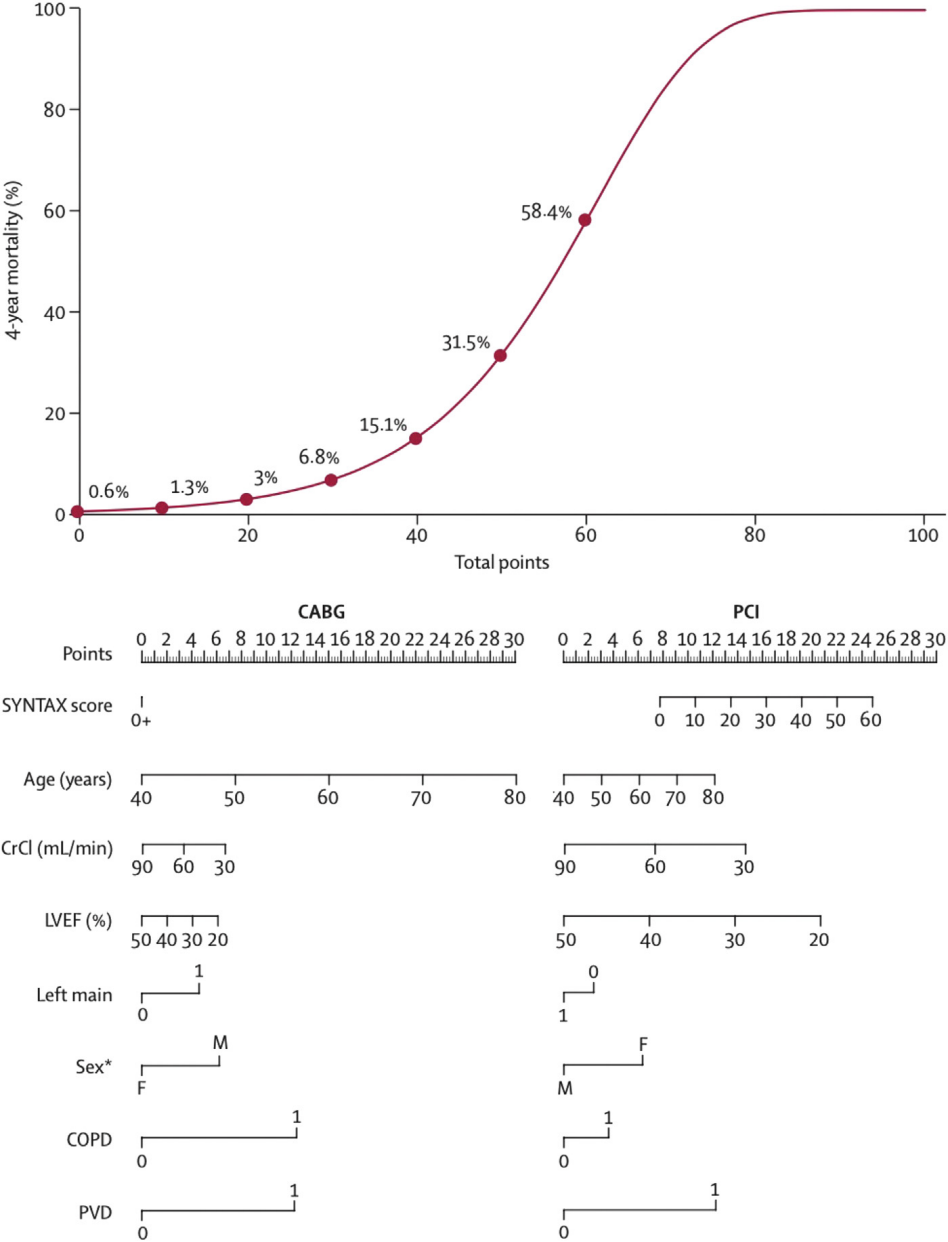
SYNTAX Score II Nomogram for Bedside Application Total number of points for 8 factors can be used to accurately predict 4-year mortality for the individual patient proposing to undergo CABG or PCI. For example, a 60-year-old man with an anatomical SYNTAX score of 30, unprotected left main coronary artery disease, creatinine clearance of 60 mL/min, an LVEF of 50%, and COPD, would have 41 points (predicted 4-year mortality 16.3%) to undergo CABG and 33 points (predicted 4-year mortality 8.7%) to undergo PCI respectively. Same example without COPD included would lead to identical points (29 points) and 4-year mortality predictions (6·3%) for CABG and PCI. Reproduced with permission from Farooq et al.^27^ ∗Because of the rarity of complex coronary artery disease in premenopausal women, mortality predictions in younger women are predominantly based on the linear relation of age with mortality. The differences in mortality predictions in younger women between CABG and PCI will therefore be affected by larger 95% CIs than those in older women. COPD = chronic obstructive pulmonary disease; CrCl = creatinine clearance; left main = unprotected left main coronary artery disease; LVEF = left ventricular ejection fraction; PVD = peripheral vascular disease; 3VD = 3-vessel disease; other abbreviations as in Figure 4.

The combination of the anatomic SXscore with the age, creatine, and LVEF (ACEF) score contained most of the prognostic information needed to predict mortality after CABG (excluding the anatomic SXscore) or PCI (including the anatomic SXscore) (Figure 13).^27^ The SSII augments the purely anatomic SXscore with anatomic and clinical factors that were shown to alter the threshold value of the anatomic SXscore for equipoise to be achieved between CABG and PCI for long-term mortality. By adopting an individualized approach using SSII it was shown that subsets of patients existed in all terciles of the SXscore in which CABG or PCI would confer a mortality benefit or offer a similar long-term prognosis. A nomogram was developed that allowed for an accurate individualized prediction of 4-year mortality in patients recommended to undergo CABG or PCI to objectively aid decision making. External validation of the SSII was performed in the multinational DELTA (Drug-Eluting Stent for Left Main CAD) registry and subsequently in the CREDO-Kyoto (Coronary REvascularization Demonstrating Outcome Study in Kyoto) registry.^27^ In addition, it was externally validated in the BEST (Bypass Surgery Versus Everolimus-Eluting Stent Implantation for Multivessel Coronary Artery Disease), PRECOMBAT (Ten-Year Outcomes of PREmier of Randomized Comparison of Sirolimus-Eluting Stent Implantation Versus Coronary Artery Bypass Surgery for Unprotected Left Main Coronary Artery Stenosis: PRE-COMBAT Trial), and EXCEL trials.

### The SYNTAX score II 2020

**Figure 14:**
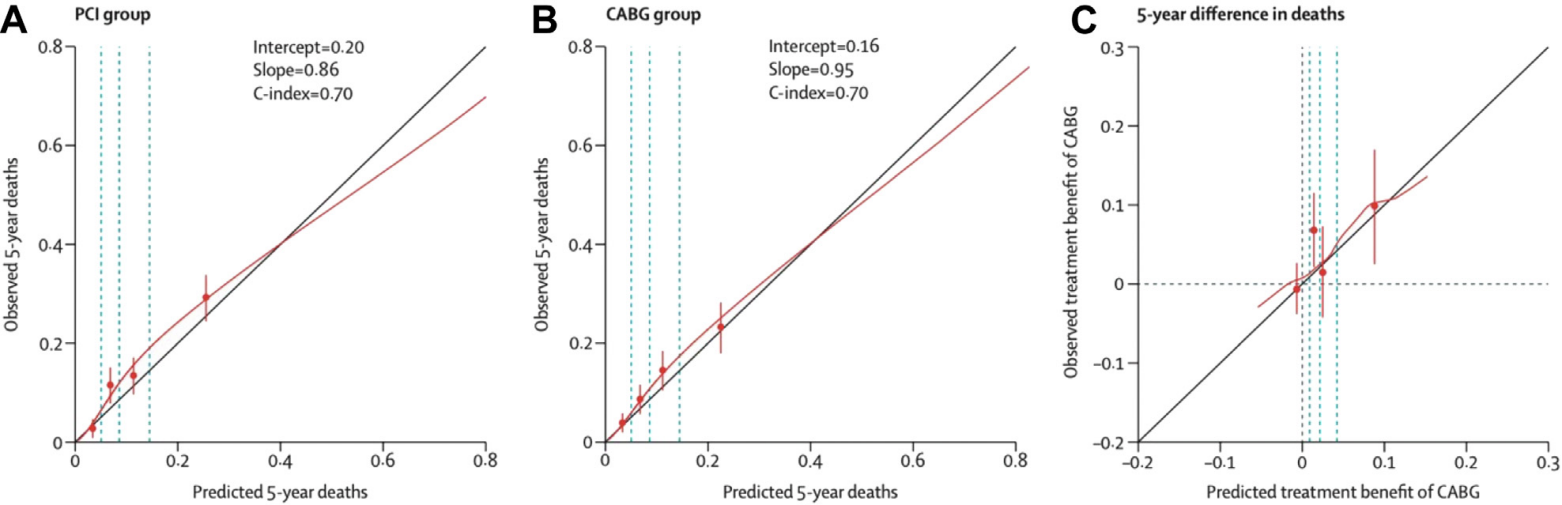
External Validation of the SYNTAX Score II 2020 Calibration plots showing the observed vs predicted 5-year deaths according to the SYNTAX score II 2020 in the PCI group **(A)** and CABG group **(B)**. **Vertical dashed lines** represent quartiles of predicted 5-year deaths. **(C)** Calibration plot showing the observed vs predicted treatment benefit of CABG, according to the SYNTAX score II 2020. **Vertical dashed lines** represent quartiles of predicted treatment benefit of CABG. Reproduced with permission from Takahashi et al.^12^ C-index = concordance index; other abbreviations as in Figure 4.

**Figure 15:**
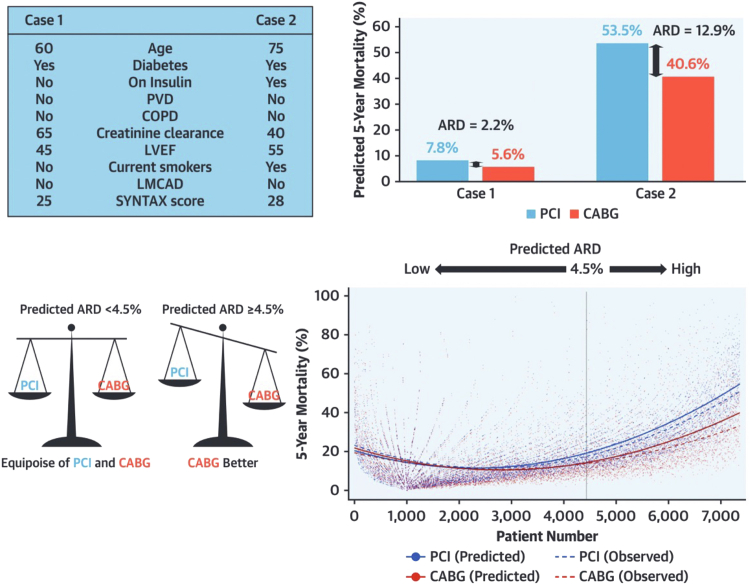
Treatment Recommendation Based on Predicted ARD for 5-Year Mortality **(Top)** In case 1, the patient has predicted 5-year mortality rates of 7.8% after PCI and 5.6% after CABG. The predicted ARD is 2.2% (<4.5%); therefore, the patient can be referred to either PCI or CABG. In case 2, the patient has predicted 5-year mortality rates of 53.5% after PCI and 40.6% after CABG. The predicted ARD is 12.9% (≥4.5%). CABG should be recommended. **(Bottom)** The individual differences between the predicted mortality **(individual scatterplots and solid smoothing curves)** as well as the observed mortality **(dashed smoothing curves)** after PCI or CABG. Reproduced with permission from Hara et al.^30^ ARD = absolute risk difference; COPD = chronic obstructive pulmonary disease; LMCAD = left main coronary artery disease; LVEF = left ventricular ejection fraction; PVD = peripheral vascular disease; other abbreviations as in Figure 4.

Long-term follow-up outcomes from the SYNTAX(ES) trial were used to re-develop the SSII, producing the SSII-2020, which can predict the 5-year risk of MACCE and 10-year risk of all-cause mortality depending on whether the treating physician intends on referring the patient for PCI or CABG.^12^ The SSII-2020 was validated using patient-level data from the FREEDOM (Future Revascularization Evaluation in Patients with Diabetes Mellitus: Optimal Management of Multivessel Disease), BEST, PRECOMBAT, EXCEL, and CREDO-Kyoto trials, all of which enrolled patients with multivessel CAD or LMCAD undergoing PCI or CABG. For 10-year all-cause mortality, the SSII-2020 had good discrimination ability for PCI and CABG (C-index 0.73 for both groups). The estimated treatment benefit of CABG over PCI varied substantially among patients in the SYNTAXES trial, and the benefit predictions were well calibrated. In the CREDO-Kyoto registry, the SSII-2020 showed helpful discrimination (C-index = 0.72; 95% CI: 0.71-0.74) and good calibration (intercept= −0.11; slope = 0.92) for predicting 5-year mortality, regardless of treatment modality (PCI or CABG), although predicted mortality rates were slightly overestimated in the highest quarter (Figure 14). The observed mortality rates after PCI and CABG were not significantly different in patients with a predicted absolute risk difference (ARD) in 5-year mortality <4.5%: observed ARD 2.1% (95% CI: −0.4% to 4.4%), whereas a significant difference in survival favoring CABG was observed in patients with a predicted ARD ≥4.5% (observed ARD: 9.7% [95% CI: 6.1%-13.3%]). This 4.5% threshold of predicted ARD effectively dichotomized patients into “equipoise of PCI and CABG” and “CABG better.” The SSII-2020 for 5-year MACE could not offer any sensible discriminating recommendation (Figure 15).^12^

### Can ML unravel unsuspected, clinically important factors predictive of long-term mortality in complex CAD? A call for “Big Data”

**Figure 16:**
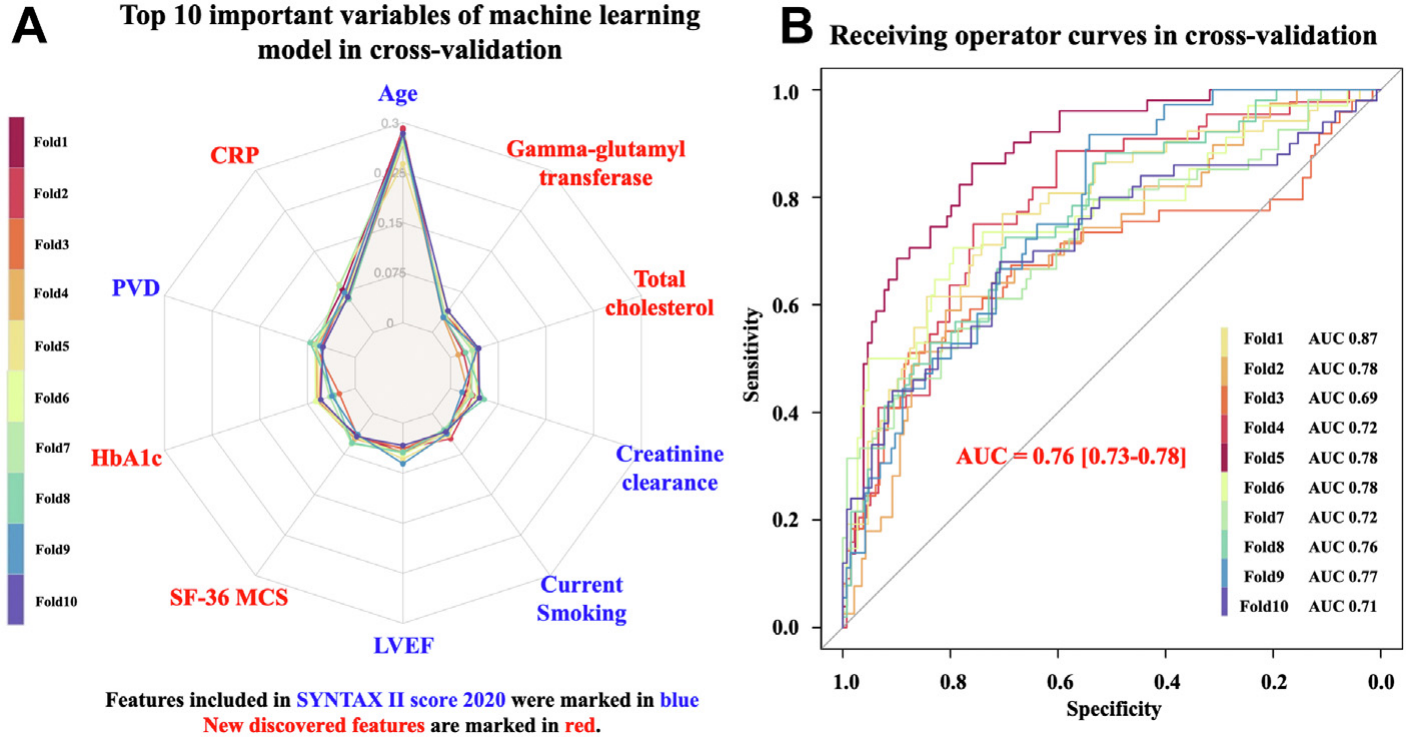
Top 10 Factors to Predict 10-Year Death in the SYNTAX Trial **(A)** Top 10 important variables disclosed by machine learning model using a 10-fold cross validation approach. **(B)** Discriminative ability for the prediction of 10-year mortality of the machine learning model using a 10-fold cross validation approach. Reproduced with permission from Ninomiya et al.^28^ AUC = area under the curve; CRP = C-reactive protein; LVEF = left ventricular ejection fraction; PVD = peripheral vascular disease; SF-36 MCS = 36-Item Short Form Survey Mental Component Summary.

Recently, ML has emerged as a novel approach for developing risk models predictive of clinical outcomes.^28^ ML and penalized regression can handle large numbers of variables and diverse parameters. Rousset et al^29^ incorporated all 428 variables in the FOURIER (Further Cardiovascular Outcomes Research With PCSK9 Inhibition in Subjects With Elevated Risk) trial to an ML model and showed that this performed better than linear regression. We used ML algorithms to predict long-term mortality following PCI or CABG with an acceptable discrimination, and an area under the curve of 0.76 in cross validation. The high-risk group classified by the ML model had significantly higher observed 10-year mortality than the intermediate (47.3% vs 18.7%, HR: 2.43; 95% CI: 2.12-2.78; *P* < 0.001) and low-risk groups (47.3% vs 10.7%, HR: 3.17; 95% CI: 2.55-3.95; *P* < 0.001) (Figure 16A).^28^ The 10 most important variables to predict 10-year mortality from the best performing model are shown in Figure 16B. Interestingly, ML algorithms identified unsuspected, but potentially important prognostic factors predictive of 10-year mortality among patients with CAD such as CRP, patient-reported preprocedural physical and mental status, Gamma-glutamyl transferase, and hemoglobin.^28^ Our ML model, which was built using clinical factors, blood sampling, imaging parameters, and patient-reported outcomes, demonstrated that those unconventional heterogeneous variables may have similar importance to conventional variables in predicting 10-year mortality. These variables are not always collected in clinical trials but may be available in large health care datasets. Using this model globally in health care systems proficient in collecting large numbers of parameters may further improve precision medicine and decision making.

## Section 5: Evidence From SYNTAXES

The SYNTAXES study provides the first randomized data, that were meticulously collected, and achieved a high follow-up rate of 93.8% for 10-year vital status. Several clinical factors were independent predictors of mortality at 10 years such as pharmacologically treated DM, increased WC, reduced LVEF, and prior CEVD and PVD. Both higher PCS and MCS were independently associated with lower 10-year mortality, with these results emphasizing the importance of routinely assessing physical and mental status in all patients before any intervention and suggesting that this should be incorporated into clinical guidelines. Similarly, several clinical factors were associated with higher 10-year mortality like having the procedure performed in western Europe and North American, current smoking, COPD, elevated CRP, anemia, and an increased HbA1c. Several factors were associated with lower mortality at 10 years such as patients being on OMT at 5 years, patients on statins, and patients who underwent on-pump CABG. Female sex and major infections both had significant impacts on 5-year mortality; however, these were no longer seen at 10 years. Nonfatal periprocedural major adverse events were an independent predictor of all-cause death in the first year postprocedure, but not at 5 or 10 years.

Several procedural factors were associated with higher all-cause mortality at 10 years like periprocedural myocardial infarction (according to SYNTAX or Fourth Universal Definition of Myocardial Infarction), extensive stenting, small stents, ≥1 heavily calcified lesion, ≥1 bifurcation lesion, a residual SYNTAX score >8, and a staged PCI. Multiple arterial grafts were associated with lower all-cause mortality at 10 years.

Other important takeaways were that patients with prior CEVD or COPD should not be precluded from revascularization, especially CABG. Also, patients who had a proximal LAD lesion did not have increased mortality at long-term follow-up, and neither did it have any impact on the treatment effects between PCI and CABG. Residual angina although common after revascularization was not associated with increased 10-year mortality.

The landmark SYNTAX trial has been a long and exciting journey, and still represents the largest (and only) assessment of revascularization with PCI or CABG in all-comers with complex CAD to date. The insights provided over this journey have had a tremendous impact on clinical practice, and interventional cardiologists and cardiothoracic surgeons are now more confident in choosing the appropriate modality of revascularization for their patients, while also being able to inform them of the risks and individualizing their treatment decisions. SYNTAX is probably among one of the few trials in the history of cardiology that has provided so many insights into the management of patients with complex CAD ranging from the impact of baseline characteristics, clinical features, procedural characteristics, and postprocedural variables. It was apparent quite early that the concept of an average treatment effect needed be replaced with individualized decision making, and indeed we have made significant progress in this area. Decision tools need to identify heterogeneity in risks between patients, the identified risks should be actionable, and must be implementable into practice.^26^ We have seen a transition from use of the anatomical SXscore to SSII to SSII-2020 when individualizing revascularization decisions between PCI and CABG in patients with complex CAD.^12^^,^^27^ The SSII-2020 has been validated in 4 randomized trials (BEST, PRECOMBAT, EXCEL, FREEDOM) and 1 large registry (CREDO-Kyoto PCI/CABG).^30^ Certainly we have seen the evolution of risk factor assessment from conventional multivariable models, to models based on the interaction for risk, to models based on calibration and treatment benefit, and now to models based on ML. Having seen this evolution, we need to promote cubic spline observed vs predicted mortality.

Notwithstanding this, the SYNTAX trial was conducted between 2005 and 2007, with a predominant use of first-generation paclitaxel-eluting stents, which may limit the generalizability of our findings to current practices**.** However, it is unavoidable that the findings derived from long-term follow-up data are based on outdated technology, whereas the evidence for contemporary technology can be derived only from short-term follow-up studies.

Having completed 10 years of the SYNTAX trial, now is the time to identify new trial targets. SYNTAX II and SYNTAX III revolution trials have already provided us with respective useful insights on how incorporating best practices in PCI improves clinical outcomes, and how the use of noninvasive physiology assessment using fractional flow reserve computed tomography (FFRCT) changes the Heart Team’s treatment decision making and procedural planning in one-fifth of patients with 3VD.^31^, ^32^, ^33^, ^34^ Several trials are in the pipeline that will change clinical practice. The ongoing FASTTRACK (A Multicenter, Pilot Study to Evaluate Safety and Feasibility Evaluation of Planning and Execution of Surgical Revascularization Solely Based on Coronary CTA and FFRCT in Patients With Complex Coronary Artery Disease) evaluates the feasibility and safety of planning and execution of CABG solely based on CT angiography combined with FFRCT without knowledge of anatomy defined by invasive angiography.^35^ The time when catheterization labs will be used only as an interventional suite is not far away. The management of complex CAD using interventional cardiology is now in its most dynamic phase ever with the technological advancements being made. With the advent of effective pharmacotherapy including anti-lipidic, anti-inflammatory, monoclonal antibodies, and microRNA and targeted therapy, it will be interesting to see how the management of complex CAD will evolve in the next 10 years.

## Section 6: Relevance of SYNTAXES to the Asian Population

The Asian population accounts for 60% of the global burden in CVD, and there has been growing evidence that there are specific differences in CAD, statin usage, risk of thrombosis, and bleeding in this population. Contemporary trials have shown that East Asian populations have a higher risk of bleeding, especially gastrointestinal bleeding and hemorrhagic stroke.^36^ Despite these observations, most guidelines are based on American or European guidelines.

Although the SYNTAX trial was conducted in Europe and North America, the trial has made significant impact in the management of complex 3VD and LM disease in the Asian population too.^12^ The trial, its design, follow-up results, scores, and prediction models developed over time will be of tremendous benefit to the Asian population and current generation trialists in Asia to implement best clinical practice and designing the trials.

There are many randomized controlled trials comparing CABG vs PCI among patients with multivessel disease or LM CAD in the Europe and Americas,^2^^,^^6^^,^^37^, ^38^, ^39^, ^40^, ^41^ and there are a paucity of trials in the Asian population. The FREEDOM trial randomized patients with diabetes and multivessel CAD and included few patients from Asia.^39^ BEST and PRECOMBAT trials were exclusively conducted in Asia.^42^^,^^43^ Although BEST randomized patients with multivessel CAD, PRECOMBAT randomized patients with unprotected LM CAD. BEST trial had 27 sites from South Korea, China, Malaysia, and Thailand. PRECOMBAT trial was conducted at 13 sites in Korea. The PRECOMBAT and BEST trials recently reported their 10-year clinical outcomes. In the CREDO-Kyoto registry, over a 3-year period (2005-2007), 15,939 consecutive patients were enrolled in 26 centers in Japan, of whom 2,981 patients were identified with 3VD (PCI: n = 1,825; CABG: n = 1,156) and SYNTAX Score II 2020 was easily applied and validated in the registry.^30^ The ongoing TUXEDO-2 (Ultra-Thin strUt Suprafex Cruz Versus Xience in a Diabetic Population With Multivessel Disease-2) randomized controlled trial will be recruiting 1,800 patients with diabetes and multivessel disease in India.^44^ The secondary objective of the study is to evaluate whether the pooled cohort of PCI provides similar clinical outcomes to CABG based on the performance goal derived from the CABG arm of the FREEDOM trial (historical cohort). The Asian population needs more trials like SYNTAX to better understand and address the inter-ethnic differences in CVD and formulate its own guidelines for CAD, which is the need of the hour.

## Funding Support and Author Disclosures

Dr Masuda has received a grant from TERUMO corporation outside the submitted work. Dr Ninomiya has received a grant from Abbott Medical Japan outside the submitted work. Dr Kotoku has received a grant for studying overseas from Fukuda Foundation for Medical Technology. Dr Serruys has received institutional grants from Sinomedical Sciences Technology, SMT (Sahajanand Medical Technologies), Philips/Volcano, GE, Xeltis, and HeartFlow, outside the submitted work. Dr Onuma has received institutional grants from Sinomedical Sciences Technology, SMT (Sahajanand Medical technologies), Philips/Volcano, GE, Xeltis, and HeartFlow, outside the submitted work. Dr Feldman is an employee of Edwards Lifesciences. All other authors have reported that they have no relationships relevant to the contents of this paper to disclose.
