# Supplementary material for: 10 Years of SYNTAX: Closing an Era of Clinical Research After Identifying New Outcome Determinants
Source: JACC Asia. 2023 May 30;3(3):409–30. doi: 10.1016/j.jacasi.2023.03.014 (PMC10308124; doi:10.1016/j.jacasi.2023.03.014)

**SUPPLEMENTAL APPENDIX**

**Post hoc analyses**

**Procedural Characteristics**

**Periprocedural Myocardial Infarction (PMI)**

The rate of PMI according to the SYNTAX and 4^th^ Universal definition of MI (UDMI), both of which required CK-MB elevation and ECG evidence of permanent myocardial damage, were respectively, 2.7% and 3.0% in the PCI arm versus 2.4% and 2.1% in the CABG arm. PMI rates according to the Society for Cardiovascular Angiography and Interventions (SCAI) or the EXCEL definition were higher with PCI compared to CABG (5.7% vs. 16.5%, p<0.001). Notably, PMIs according to the SYNTAX and 4^th^ UDMI were more strongly associated with mortality than PMIs according to EXCEL and SCAI, which relied solely on isolated enzyme elevations. The impact of these “enzyme-driven events” on time-to-event curves and composite endpoints was greater in the surgical cohort. PMIs after PCI were associated with 10-year mortality regardless of definition, whereas their impact on mortality after CABG was limited to the first year. PMIs after PCI and CABG were both independent predictors for all-cause mortality at 1-year, whilst only those after PCI remained independent predictors for all-cause mortality at 10 years. In the overall cohort, PMIs according to the SYNTAX or 4^th^ UDMI were significantly associated with all-cause mortality at 1- (31% and 30.2%) and 10-years (53.4% and 49.6%), whereas the association failed to be significant with the SCAI and EXCEL definitions at 10 years (31.1%) and was low at 1 year (10.6%) (Supplementary Figure 1).^1^

**Impact of stent length and diameter**

Total stent length (TSL) was defined as the sum of all stent lengths implanted per patient, whilst average stent diameter (ASD) was the average diameter of all stents implanted per patient. Not extensive stenting was defined as TSL≤100mm and small stenting as ASD ≤3mm. Patients treated with extensive stenting and small stents had higher rates of 3VD, lesion length of >20 mm, bifurcation lesions, and higher SXscores. TSL as a continuous variable was significantly associated with 10-year mortality (aHR, 1.05 [1.01-1.09] per 10mm increase). PCI with extensive stenting had a higher 10-year mortality than CABG (aHR, 1.94[1.36-2.77]) and PCI with not-extensive stenting (aHR, 1.94[1.36-2.77]). PCI with small stents was associated with higher 10-year mortality compared to CABG (aHR, 1.66[1.23-2.26]) and PCI performed with large stents (aHR, 1.74[1.19-2.53]). Patients treated with non-extensive and large stents had similar mortality rates (24% versus 23.8%) to those treated with CABG. The mortality outcome of PCI without extensive stenting was similar to CABG (aHR, 1.01[0.75-1.36]). In patients with 3VD, extensive stenting PCI was associated with higher 10-year mortality compared to CABG and not extensive stenting (33.0, 25.9, and 20.5% respectively; Log-rank p=0.001, Supplementary Figure 2).^2^

**Single or multiple arterial bypass graft surgery versus PCI**

From the study cohort, 1743 patients received revascularization with PCI (n=901, 51.7%), or CABG with a single arterial graft (SAG, n=532, 30.5%) or multiple (≥2) arterial grafts (MAG, n=310, 17.8%). At maximum follow-up, all-cause death occurred in 305 (33.9%), 175 (32.9%), and 70 (22.6%) patients in the PCI, SAG, and MAG groups respectively (P<0.001). MAG (aHR, 0.66[0.49-0.89]), but not SAG (aHR, 0.83(0.67-1.03]), was associated with significantly lower all-cause mortality compared with PCI. In patients with 3VD, both MAG (aHR, 0.55[0.37-0.81]) and SAG (aHR, 0.68[0.50-0.91]) were associated with significantly lower mortality than PCI, whereas in LMCAD patients, there were no significant differences between the three groups. In patients with revascularization of all three major myocardial territories, a positive correlation was observed between the number of myocardial territories receiving arterial grafts and survival (P_trend_=0.003). Diabetic patients who underwent CABG with MAG had numerically lower mortality than SAG or PCI, whilst those who received SAG had numerically higher mortality than those who underwent PCI. In the non-DM population, both MAG and SAG were associated with significantly lower all-cause mortality than PCI (Supplementary Figure 3).^3^

**CABG with multiple versus single arterial grafts**

The total treated CABG population in the SYNTAX trial compromised of 1466 patients (randomised CABG cohort n=831, randomised PCI cohort n=11, CABG registry n=624), of whom 1001 (68.3%) received a SAG, which was a left internal thoracic artery (ITA) in 99.4%, whilst 465 (31.7%) received MAG, with bilateral ITA grafting in 341 patients (73.3%). MAG in patients with 3VD was associated with significantly lower all-cause death at 12.6 years follow-up (aHR 0.65[0.44-0.97], p=0.033). Other subgroups that were prespecified in the SYNTAX trial such as LMCAD and those with and those without DM had a non-significant trend towards better survival at the 12.6-year follow-up mark following the use of MAG (Supplementary Figure 4).^4^

**Impact of On-pump and Off-pump Coronary Artery Bypass Grafts**

The heterogeneous use of on- and off-pump CABG was described using the United Nations geoscheme to sub-divide countries and regions, and this showed substantial inter-site variation in the use of off-pump CABG despite baseline characteristics being largely homogeneous amongst the three groups. Data suggest that the long-term effects of off-pump CABG are inferior to on-pump CABG, with the main reasons being lower rates of CR and reduced graft patency, particularly when off-pump surgery is performed by surgeons with limited experience. MACCE at 5 years was significantly lower following on-pump CABG compared to PCI, whilst it was only numerically lower with off-pump CABG compared to PCI. The cumulative incidence of crude 10-year mortality was significantly lower with on-pump CABG compared to PCI, and comparable between off-pump CABG and PCI.

**CAD involving the proximal left anterior descending artery**

The optimal mode of revascularization for lesions in the proximal left anterior descending artery (P-LAD) remains unclear, especially when associated with 3VD. Among patients with 3VD the presence of a P-LAD lesion was not associated with a higher incidence of MACCE at 5 years or all-cause death at 10 years, and furthermore there was no evidence that the presence of a P-LAD lesion impacted on the treatment effects of PCI and CABG.^5^

**Patients with Heavy Calcification**

Approximately a third of the cohort had at least one heavily calcified lesion (HCL), defined as radiopacities observed without cardiac motion and before contrast injection, generally compromising both sides of the arterial lumen, in vessels ≥1.5mm in diameter with >50% diameter stenosis. Of note, the study did not consider the presence of heavy calcification in segments with non-obstructive CAD (<50% diameter stenosis). Patients with ≥1 HCL had a higher crude mortality rate at 10 years than those without (36.4% vs 22.3%; HR:1.79[1.49-2.16]; p<0.001, Supplementary Figure 5A), with HCL an independent predictor of 10-year mortality (HR: 1.36[1.09-1.69]; P=0.006). There was a significant interaction for mortality between the treatment effect (PCI and CABG) and the presence or absence of HCLs (P_interaction_=0.005). In patients without HCLs, mortality was significantly higher after PCI than after CABG (26.8% vs 18.8%, p=0.003), whereas in those with HCLs, there was no significant difference (34.0% vs 39.0%, p=0.264, Supplementary Figure 5B). In the CABG arm, patients with ≥2 HCLs had higher mortality than those with 1 HCL, whereas, in the PCI arm, there was no significant difference between patients with 1 or ≥2 HCLs.^6^

**Patients with Bifurcation lesions**

The SYNTAX cohort including 1300 patients with ≥1 bifurcation and 487 patients with no bifurcations. Among patients treated with PCI, those with ≥1 bifurcation lesion had a significantly higher risk for all-cause death (19.8% vs. 30.1%; p=0.007), whereas following CABG, mortality was similar in patients with and those without bifurcation lesions (23.3% vs 23.0%; p=0.021). According to the SSII-2020, among those with ≥1 bifurcation, there was equipoise for all-cause mortality between PCI and CABG in 2 quartiles of the population, whereas CABG was superior to PCI in the 2 remaining quartiles. There was no significant interaction between the presence of a bifurcation lesion and the treatment effect on all-cause mortality at 5 years following PCI or CABG. In the PCI arm, the risk of repeat revascularization at 5 years was significantly higher among patients with bifurcations than without, whereas there was no significant difference following CABG. At 10 years, the presence of ≥1 bifurcation lesion was an independent predictor of all-cause death with PCI, whereas mortality following CABG was similar between patients with and without bifurcation lesions. Landmark analysis in patients with bifurcation lesions treated with PCI showed a continuous and significant divergence of cumulative mortality beyond 5 years. In patients with 3VD and ≥1 bifurcation, CABG had an average beneficial effect on survival at 10 years compared with PCI, whereas in patients with distal LMCAD, the risk for all-cause mortality was comparable. The risk of repeat revascularization at 5 years was significantly higher among patients receiving a 2-stent compared with a 1-stent technique. At 10 years, the use of a 2-stent technique was an independent predictor of all-cause death (Supplementary Figure 6).^7^

**Patients with total coronary artery occlusions**

In SYNTAX, the 460 patients who had at least one total occlusion (TOs), which included all occluded vessels irrespective of their duration, had comparable 10-year mortality to those patients with no TO (27.6% vs. 26.1%; HR:1.06; P=0.583). In patients with TOs, the status of recanalization or revascularization was not associated with 10-year all-cause mortality, and this was irrespective of the assigned treatment. The status of recanalization/revascularization of TOs in the LM and/or LAD artery did not have an impact on mortality. When patients were stratified according to the presence or absence of a TO, there was no difference between PCI and CABG in terms of 10-year mortality (Supplementary Figure 7).^8^

**Completeness of revascularization**

Complete revascularization (CR) was a binary outcome, which was defined by revascularization of all lesions with ≥50% diameter stenosis in vessels ≥1.5mm. Quantitively a residual SXscore (rSS) of 0 implies CR, whereas an rSS>0 identifies a degree of incomplete revascularization (IR). IR was more frequent in patients having PCI versus CABG (56.6% vs. 36.8%) and more common in those with 3VD than LMCA disease following both PCI (58.5% vs. 53.8%) and CABG (42.8% vs. 27.5%). There was no significant difference in 10-year mortality between patients undergoing PCI who had CR and those undergoing CABG with either IR/CR. Patients with IR had a significantly higher risk of all-cause death at 10 years compared with CABG and CR. When patients with PCI were stratified according to rSS, those with an rSS≤8 had no significant difference in all-cause death at 10 years compared to the other terciles, whereas an rSS>8 resulted in a significantly higher risk of 10-year all-cause death versus those undergoing PCI with CR (50.1% vs. 22.2%; aHR, 3.40[2.13-5.43]). PCI patients with IR had higher anatomic SXscores and consequently more complex lesions. The adjusted risk (aHR) of 10-year all-cause death was significantly higher in patients with PCI and IR compared with CABG and CR (aHR 1.70[1.20-2.42]). Patients with an rSS of >8 had a 3-fold higher risk of 10-year all-cause death compared with those with an rSS of 0 (Supplementary Figure 8).^9^

**Staged percutaneous revascularization**

Staged PCIs (SPCI) were allowed within 72 hours of the index procedure, or if renal insufficiency or contrast-induced nephropathy occurred, within 14 days. Patients who underwent SPCI had higher 10-year mortality compared to those who did not (40% vs. 26.6%; HR 1.69; P<0.01) and those having CABG (40% vs. 24.5%; HR 1.85; P<0.01, Supplementary Figure 9) with possible reasons their more complex CAD, their lower rate of CR, and their higher rSS.^10^

**Post-Procedural Variables**

**Optimal Medical Therapy (OMT)**

OMT, which was defined as taking the combination of 4 types of medications: at least 1 antiplatelet drug, statin, angiotensin converting enzyme inhibitor (ACEI)/ angiotensin receptor blocker (ARB) and beta-blocker, was being taken by 46.1% of patients at 5-years, and this resulted in significantly lower mortality at 10 years compared with those on ≤2 types of medications (p=0.002). Patients undergoing CABG who were taking anti-platelet drugs and statins at 5 years had lower 10-year mortality than those who weren’t. From the SYNTAXES study it is evident that ≥3 types of OMT medications should be maintained for 5 years following revascularization (Supplementary Figure 10).^11^

**Major infections**

Periprocedural major infections were defined as major infections within 60 days of the index procedure. Despite the initial association between major infections and 5 year mortality, there was no significant difference in 10-year mortality between patients who had postprocedural major infections and those who did not (30.8% versus 24.5%; p=0.057), suggesting that the impact of major infection on mortality subsided beyond 5 years ^12^.

**Impact of residual angina**

The incidence of residual angina (RA), which was defined by a self-reported SAQ-AF scale ≤90 at 1-year follow-up post revascularization, was 26.1%, and this was independently associated with repeat revascularization at 5 years (18.3% vs 11.5%; aHR: 1.54; 95% CI: 1.10-2.15) – driven mainly by the PCI arm; however this did not significantly increase 10-year mortality (22.1 vs. 21.6%; aHR: 1.11; 95% CI 0.83-1.47), irrespective of the primary modality of revascularization or the severity of RA.^13^ A lower SAQ-AF score at 1 year was associated with a higher risk of repeat revascularization at 5 years^13^ (Supplementary Figure 11).

**Impact of Peri-procedural Major Adverse Events (PMAE)**

Peri-procedural major adverse events (PMAE) were defined as the occurrence within 30 days post-procedure of death, periprocedural and spontaneous MI, ischemic and hemorrhagic stroke, repeat percutaneous or surgical revascularization, major infection, stent thrombosis/ graft occlusion, bleeding, major arrhythmia, heart failure, acute respiratory failure, acute renal failure, and wound dehiscence. Non-fatal PMAEs occurred less frequently following PCI than CABG (11.2% vs. 28.2%; p<0.001) and were an independent predictor of all-cause mortality in the first-year post procedure, but not at 5- or 10-years, in either treatment modality. Importantly within 30 days of the procedure, patients treated with PCI had fewer strokes, major infections, major arrhythmias, acute respiratory failure, and acute renal failure compared to those treated with CABG. The presence of PMAEs at one month may therefore serve as a trigger for physicians to consider more frequent follow-up and aggressive adjunctive pharmacological therapy to amend or prevent the recurrence of adverse events for at least a year.

References:

1. Hara H, Serruys PW, Takahashi K et al. Impact of Peri-Procedural Myocardial Infarction on Outcomes After Revascularization. J Am Coll Cardiol 2020;76:1622-1639.

2. Hara H, Ono M, Kawashima H et al. Impact of stent length and diameter on 10‐year mortality in the SYNTAXES trial. Catheterization and Cardiovascular Interventions 2021;98:E379-E387.

3. Davierwala PM, Gao C, Thuijs DJ et al. Single or multiple arterial bypass graft surgery vs. percutaneous coronary intervention in patients with three-vessel or left main coronary artery disease. European heart journal 2022;43:1334-1344.

4. Thuijs D, Davierwala P, Milojevic M et al. Long-term survival after coronary bypass surgery with multiple versus single arterial grafts. Eur J Cardiothorac Surg 2022;61:925-933.

5. Ono M, Hara H, Gao C et al. Mortality after multivessel revascularisation involving the proximal left anterior descending artery. Heart 2022;108:1784-1791.

6. Kawashima H, Serruys PW, Hara H et al. 10-Year All-Cause Mortality Following Percutaneous or Surgical Revascularization in Patients With Heavy Calcification. JACC Cardiovasc Interv 2022;15:193-204.

7. Ninomiya K, Serruys PW, Garg S et al. Predicted and Observed Mortality at 10 Years in Patients With Bifurcation Lesions in the SYNTAX Trial. JACC Cardiovasc Interv 2022;15:1231-1242.

8. Kawashima H, Takahashi K, Ono M et al. Mortality 10 Years After Percutaneous or Surgical Revascularization in Patients With Total Coronary Artery Occlusions. J Am Coll Cardiol 2021;77:529-540.

9. Takahashi K, Serruys PW, Gao C et al. Ten-Year All-Cause Death According to Completeness of Revascularization in Patients With Three-Vessel Disease or Left Main Coronary Artery Disease: Insights From the SYNTAX Extended Survival Study. Circulation 2021;144:96-109.

10. Kawashima H, Ono M, Hara H et al. Ten-Year All-Cause Mortality Following Staged Percutaneous Revascularization in Patients With Complex Coronary Artery Disease. Cardiovasc Revasc Med 2022;38:124-126.

11. Kawashima H, Serruys PW, Ono M et al. Impact of Optimal Medical Therapy on 10-Year Mortality After Coronary Revascularization. J Am Coll Cardiol 2021;78:27-38.

12. Ono M, Kawashima H, Hara H et al. Impact of major infections on 10-year mortality after revascularization in patients with complex coronary artery disease. Int J Cardiol 2021;341:9-12.

13. Ono M, Serruys PW, Kawashima H et al. Impact of residual angina on long-term clinical outcomes after percutaneous coronary intervention or coronary artery bypass graft for complex coronary artery disease. Eur Heart J Qual Care Clin Outcomes 2022.

**Supplemental Figure 1. MACCE Rates According to Periprocedural MI Definitions**

(A to E) Major adverse cardiac or cerebrovascular event (MACCE) rates in the present study population (n=1,652) according to the (A) SYNTAX, (B) Fourth UDMI, (C) ISCHEMIA, and (D) SCAI or EXCEL definitions. The rates of MACCE were calculated using the Kaplan-Meier method. (E) MACCE rates excluding PMI. (F) Rates of all-cause death. The rates of all-cause death were calculated using the Kaplan-Meier method. Reproduced with permission from Hara H, J Am Coll Cardiol 2020.^1^CABG = coronary artery bypass grafting surgery; CK = creatine kinase; CK-MB = creatine kinase-myocardial band; PCI = percutaneous coronary intervention; ULN = upper limit of normal


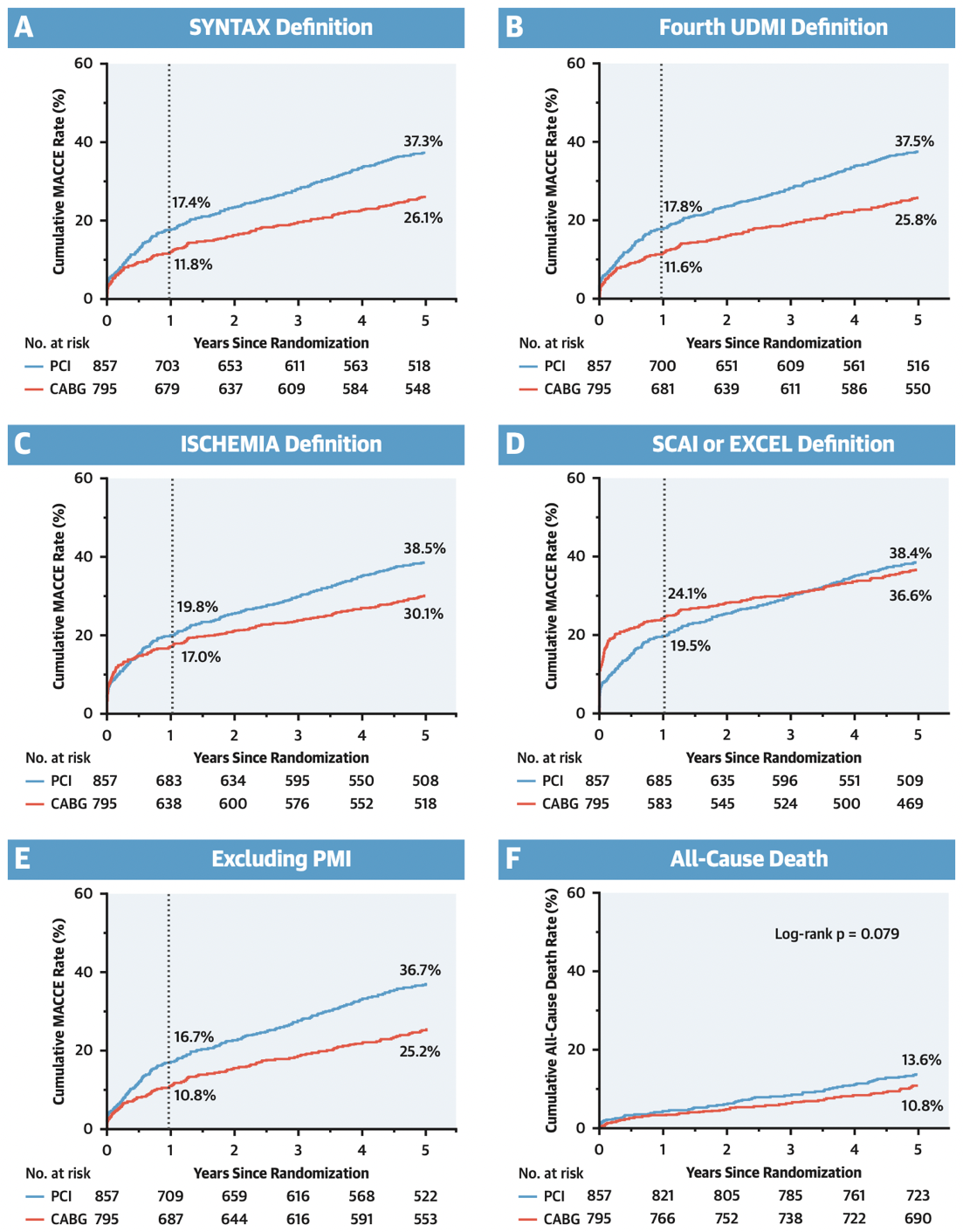


**Supplemental Figure 2. All-cause mortality at 10 years in patients with or without extensive stenting.**

(a–c) Mortality rates in patients with extensive stenting PCI, not-extensive stenting PCI and CABG. (a) The overall cohort. (b) Three-vessel disease (3VD) cohort. (c) Left main coronary artery disease (LMCAD) cohort. (d–f) Mortality rates in patients with small stenting PCI, large stenting PCI and CABG. (d) The overall cohort. (e) 3VD cohort. (f) LMCAD cohort. Reproduced with permission from Hara H, Catheterization and Cardiovascular Interventions 2021.^2^ CABG = coronary artery bypass grafting surgery; PCI = percutaneous coronary intervention

**
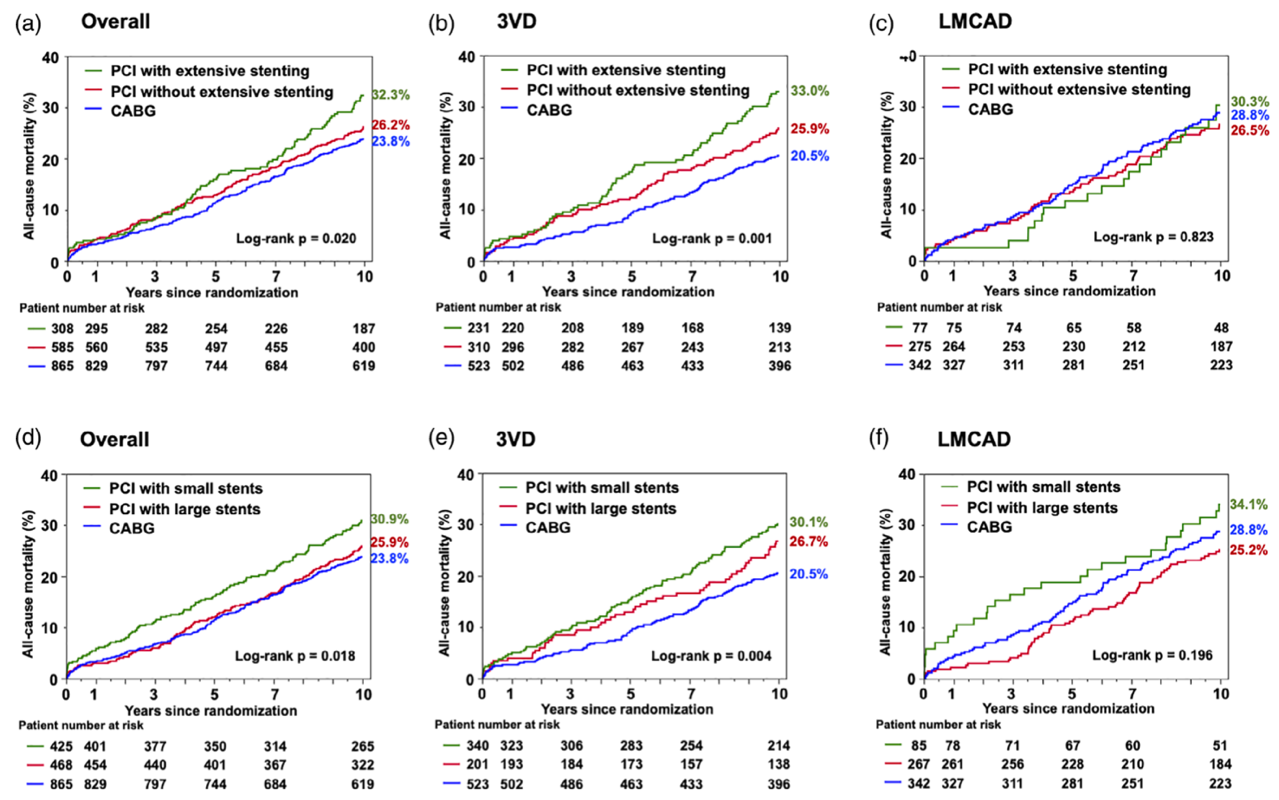
**

**Supplemental Figure 3. All-cause mortality in patients receiving SAG or MAG versus PCI:**

(A) left main coronary artery disease; (B) three-vessel disease; (C) diabetes mellitus; (D) non-diabetes mellitus; (E) SYNTAX score _33. Reproduced with permission from Davierwala PM, European heart journal 2022.^3^ 3VD = three-vessel disease; DM = diabetes mellitus; LM = left main; MAG = multiple arterial grafting; PCI = percutaneous coronary intervention; SAG = single arterial grafting

**
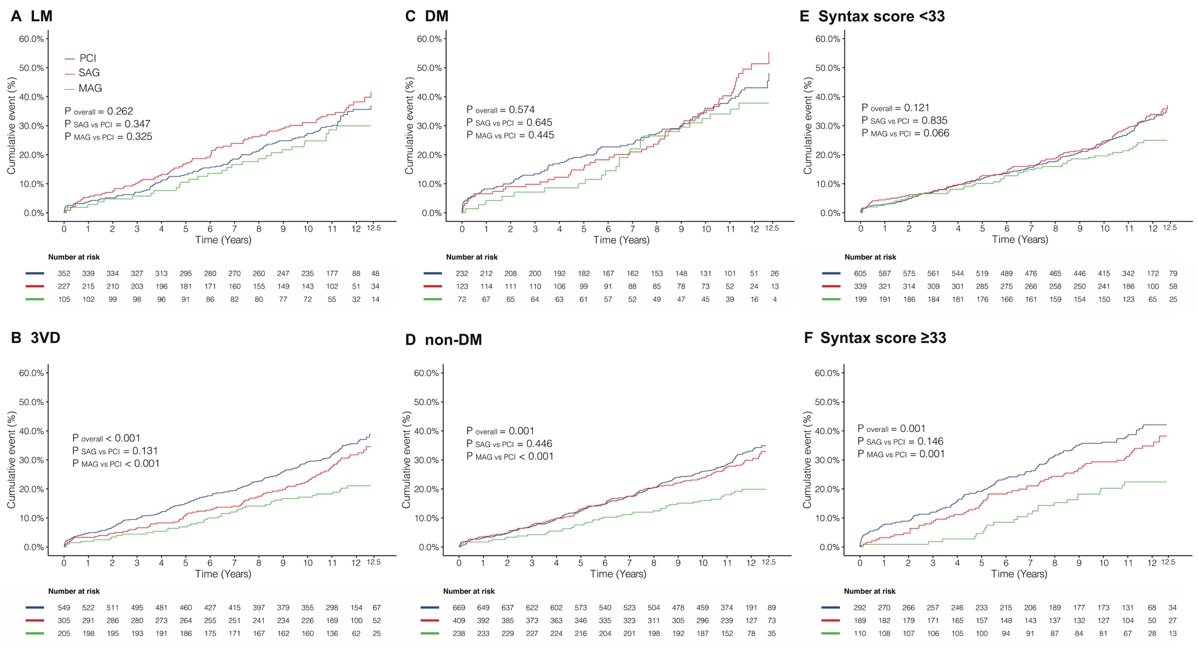
**

**Supplemental Figure 4. Mortality in SAG and MAG patients in specific clinically important subgroups at maximum follow-up**

(A) 3VD, (B) LMCAD, (C) preoperative diabetes mellitus and (D) no preoperative diabetes mellitus. Reproduced with permission from Thuijs D, Eur J Cardiothorac Surg 2022.^4^

3VD = three-vessel disease; CI = confidence interval; LMCAD = left main coronary artery disease; MAG = multiple arterial grafts; SAG = single arterial graft. The axis label ‘years since treatment allocation’ indicates a follow-up period starting from the day of randomization or allocation to percutaneous coronary intervention-ineligible coronary artery bypass grafting registry, which does not necessarily correlate to the operative day

**
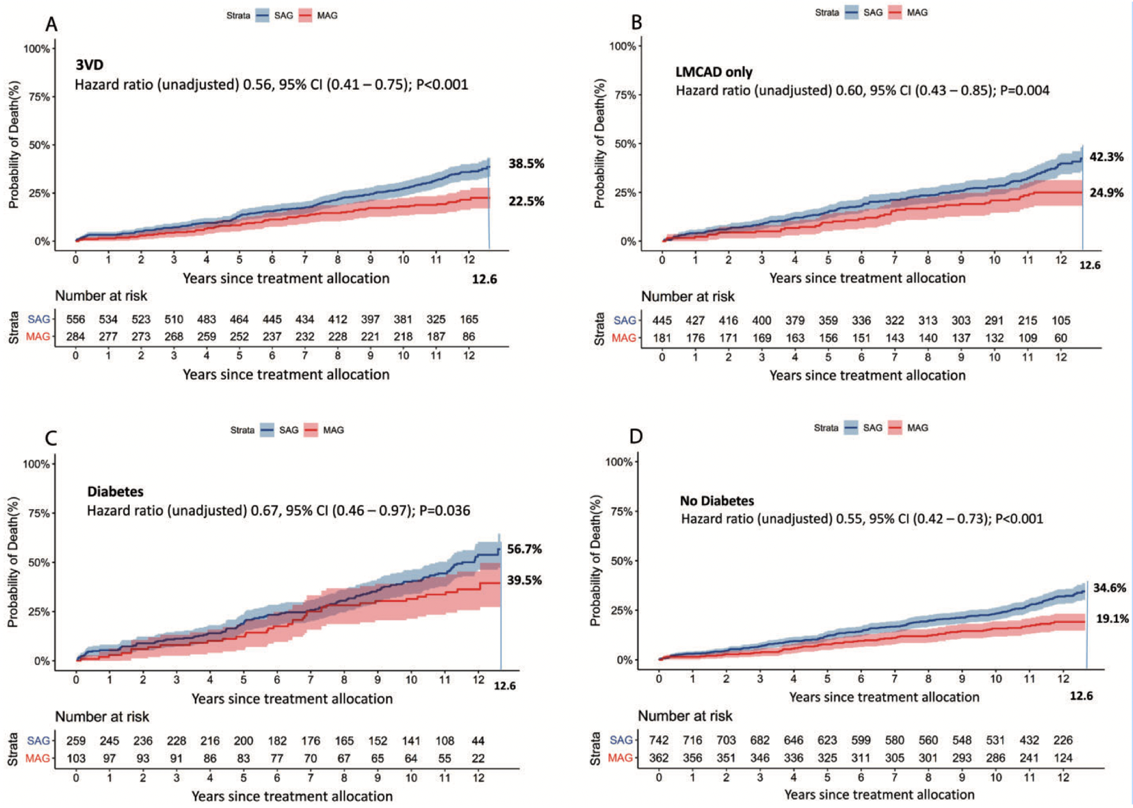
**

**Supplemental Figure 5. 10-year all-cause mortality in patients with or without heavy calcification**

1. Kaplan–Meier curves for all-cause mortality at 10 years according to the presence of at least one HCL (N=1,800). (B) Kaplan–Meier curves for all-cause mortality at 10 years presence of at least one HCL and randomized treatment with PCI or CABG. Reproduced with permission from Kawashima H, JACC Cardiovasc Interv 2022.^6^ HCL = Heavily calcified lesion

**
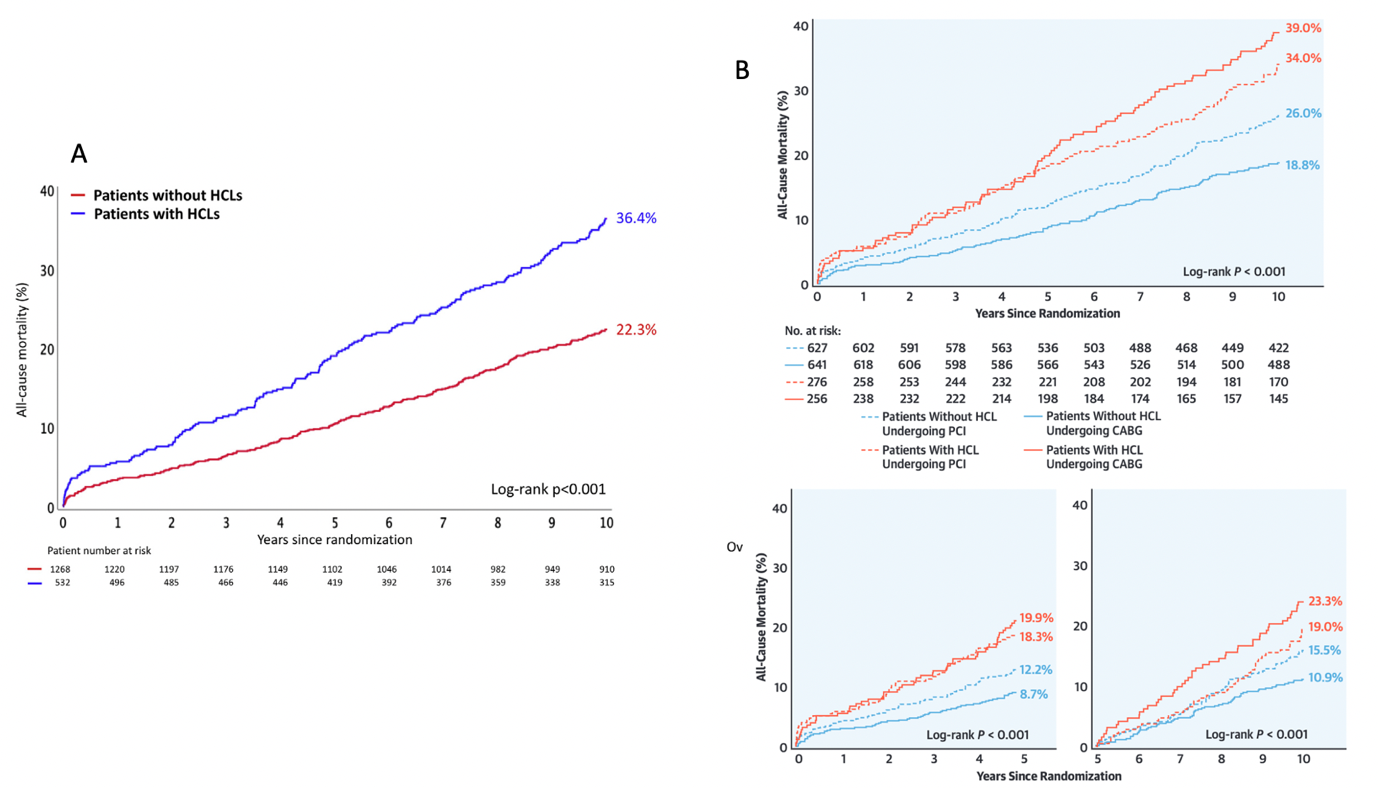
**

**Supplemental Figure 6. All-cause mortality at 10 years with respect to the bifurcation lesions**

In the PCI arm, the incidence of all-cause death at 10 years was significantly higher in patients with > 1 bifurcation, whereas the mortality in the CABG arm was similar. The landmark analysis shows that in patients with bifurcation lesions treated with PCI, there was a continuous and significant divergence in the cumulative incidence of mortality beyond 5 years. Reproduced with permission from Ninomiya K, JACC Cardiovasc Interv 2022.^7^ 3VD = 3-vessel disease; CABG = coronary artery bypass grafting; LMCAD = left main coronary artery disease; PCI = percutaneous coronary intervention; SYNTAX = Synergy between PCI with Taxus and Cardiac Surgery

**
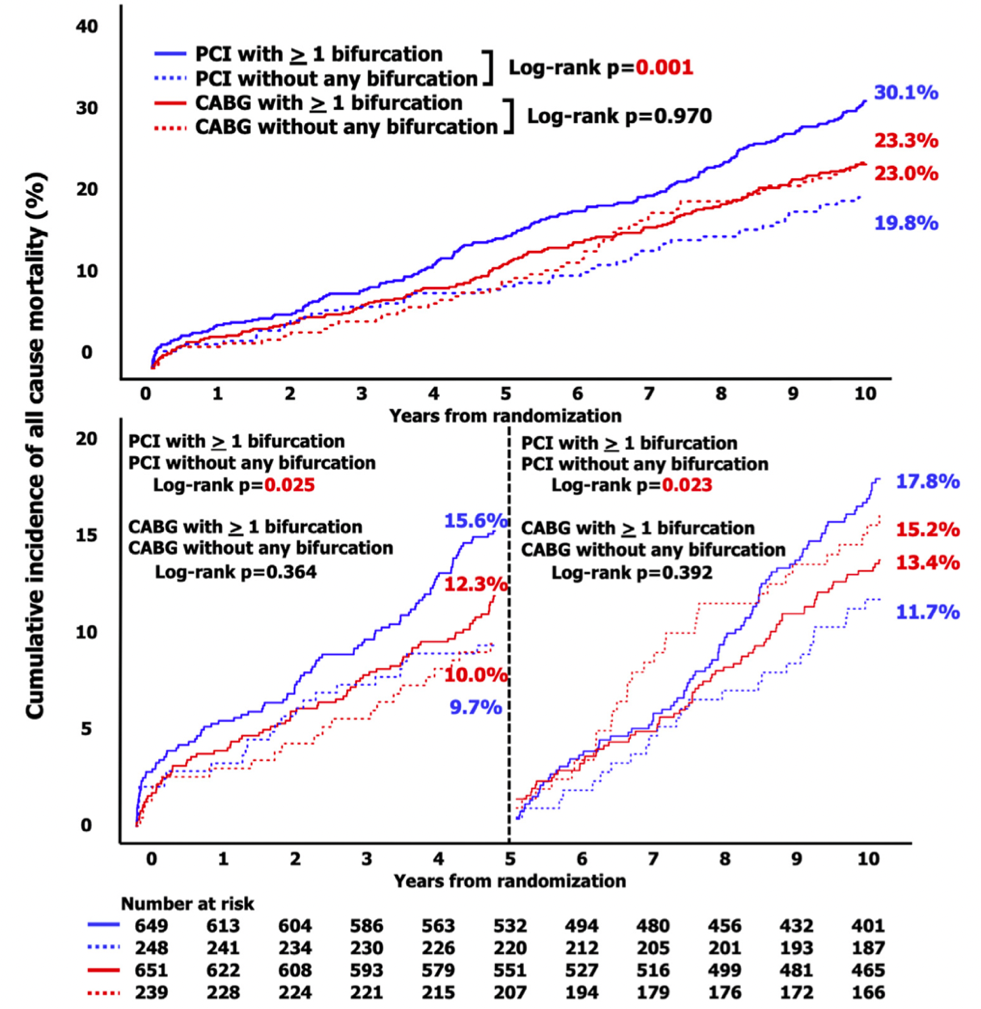
**

**Supplemental Figure 7. All-Cause Mortality at 10 Years According to the Status of Total Occlusion Recanalization or Revascularization**

Kaplan-Meier curves of all-cause mortality at 10 years according to the status of total occlusion (TO) recanalization or revascularization and randomized treatment of percutaneous coronary intervention (PCI) or coronary artery bypass grafting (CABG) (TO population in SYNTAXES [Synergy Between PCI With Taxus and Cardiac Surgery Extended Survival] study, n = 460). TO recanalization–PCI (blue line) versus non-TO recanalization–PCI (red dotted line) versus TO revascularization–CABG (grey line) versus non-TO revascularization–CABG (purple dotted line). Reproduced with permission from Kawashima H, J Am Coll Cardiol 2021.^8^


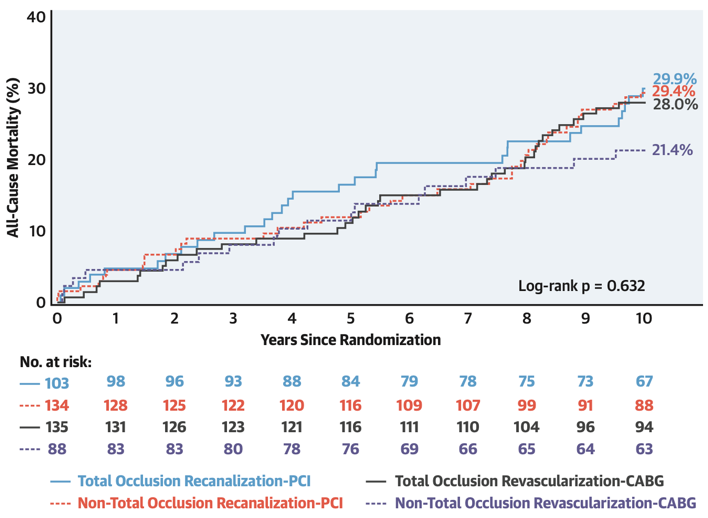


**Supplemental Figure 8. All-cause death at 10 years with respect to the completeness of revascularization**.

A, Overall population. B, 3VD cohort. C, LMCAD cohort. Reproduced with permission from Takahashi K, Circulation 2021.^9^ 3VD = 3-vessel disease; CABG = coronary artery bypass grafting; CR = complete revascularization; HR = hazard ratio; IR = incomplete revascularization; LMCAD = left main coronary artery disease; and PCI = percutaneous coronary intervention.

**
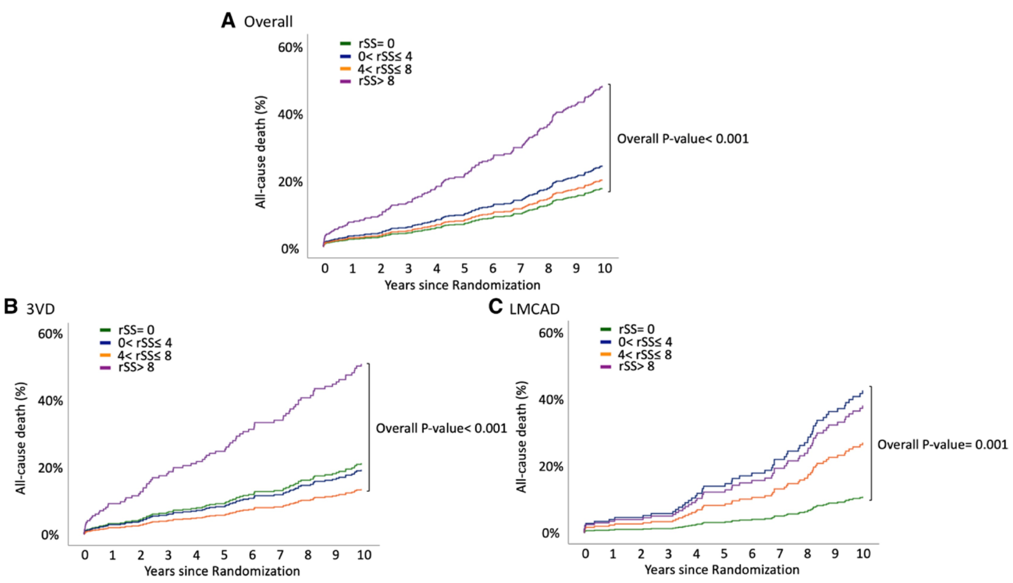
**

**Supplemental Figure 9. All-cause mortality at 10 years with respect to staged PCI**

The HRs comparing patients with SPCI (blue) versus those without (red) and patients with SPCI versus patients post CABG (green). Reproduced with permission from Kawashima H, Cardiovasc Revasc Med 2022.^10^ SPCI = staged percutaneous coronary intervention; CABG = coronary artery bypass grafting; HR = hazard ratio; CI = confidence interval.

**
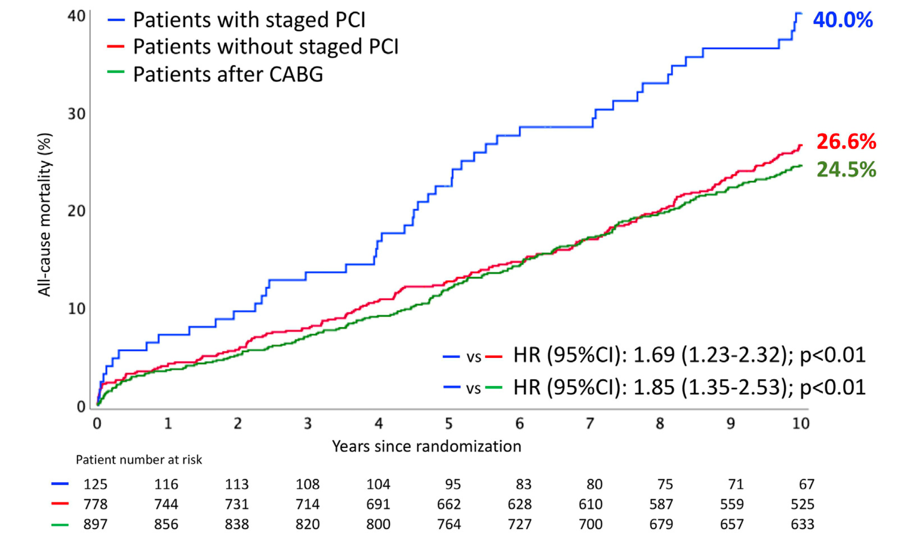
**

**Supplemental Figure 10.** **All-Cause Mortality from 5 to 10 Years with respect to optimal medical therapy**

The Kaplan-Meier curves were stratified according to the number of individual optimal medical therapy agents at 5 years. Patients on none, 1, or 2 types of medications (blue line) versus patients on 3 types of medications (red line) versus patients on 4 types of medications (black line). Reproduced with permission from Kawashima H, J Am Coll Cardiol 2021.^11^


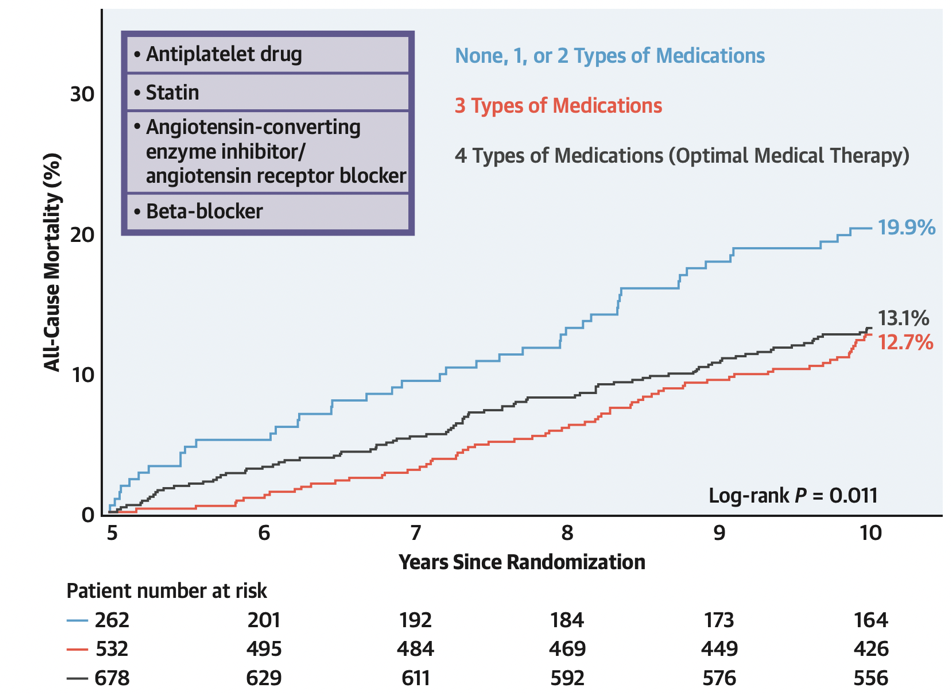


**Supplemental Figure 11. All-cause death at 10 years in patients with or without patient-reported angina at 1-year visit**.

There was no significant difference in incidence of all-cause death up to 10 years between angina and no-angina at a 1-year visit. Reproduced with permission from Ono M, Eur Heart J Qual Care Clin Outcomes 2022.^13^


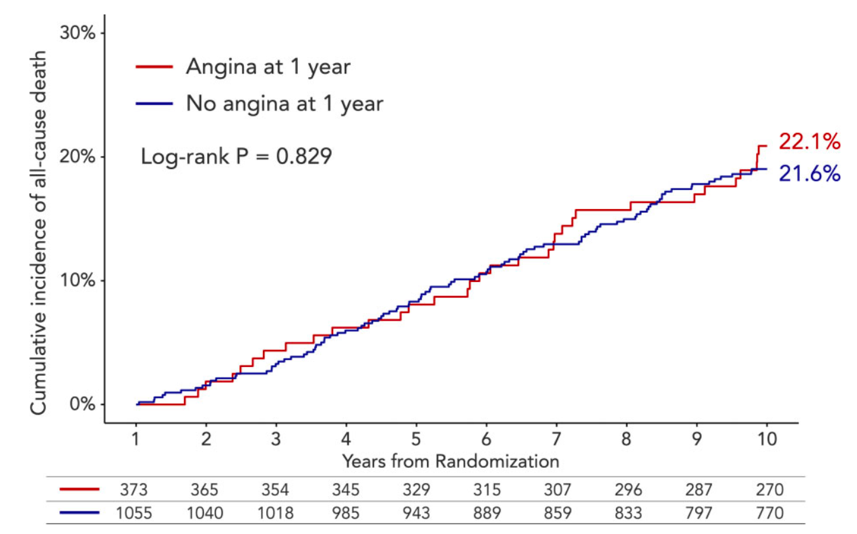

Supplement: Supplemental Material [file mmc1.docx]
